# Targeting Class I Histone Deacetylases in Human Uterine Leiomyosarcoma

**DOI:** 10.3390/cells11233801

**Published:** 2022-11-27

**Authors:** Qiwei Yang, Ali Falahati, Azad Khosh, Hanaa Mohammed, Wenjun Kang, Ana Corachán, Maria Victoria Bariani, Thomas G. Boyer, Ayman Al-Hendy

**Affiliations:** 1 Department of Obstetrics and Gynecology, University of Chicago, Chicago, IL 60637, USA; 2 Department of Biology, Yazd University, Yazd 891581841, Iran; 3 Anatomy Department, Faculty of Medicine, Sohag University, Sohag 82524, Egypt; 4 Center for Research Informatics, University of Chicago, Chicago, IL 60637, USA; 5 Department of Paediatrics, University of Valencia, Obstetrics and Gynecology, 46026 Valencia, Spain; 6 Department of Molecular Medicine, Institute of Biotechnology, University of Texas Health Science Center at San Antonio, San Antonio, TX 78229, USA

**Keywords:** uterine leiomyosarcoma, leiomyoma, histone deacetylase, inhibitors, transcriptome analysis, apoptosis, cell cycle, EMT, histone modifications, transcription factors, miRNAs

## Abstract

Uterine leiomyosarcoma (uLMS) is the most frequent subtype of uterine sarcoma that presents a poor prognosis, high rates of recurrence, and metastasis. Currently, the molecular mechanism of the origin and development of uLMS is unknown. Class I histone deacetylases (including HDAC1, 2, 3, and 8) are one of the major classes of the HDAC family and catalyze the removal of acetyl groups from lysine residues in histones and cellular proteins. Class I HDACs exhibit distinct cellular and subcellular expression patterns and are involved in many biological processes and diseases through diverse signaling pathways. However, the link between class I HDACs and uLMS is still being determined. In this study, we assessed the expression panel of Class I HDACs in uLMS and characterized the role and mechanism of class I HDACs in the pathogenesis of uLMS. Immunohistochemistry analysis revealed that HDAC1, 2, and 3 are aberrantly upregulated in uLMS tissues compared to adjacent myometrium. Immunoblot analysis demonstrated that the expression levels of HDAC 1, 2, and 3 exhibited a graded increase from normal and benign to malignant uterine tumor cells. Furthermore, inhibition of HDACs with Class I HDACs inhibitor (Tucidinostat) decreased the uLMS proliferation in a dose-dependent manner. Notably, gene set enrichment analysis of differentially expressed genes (DEGs) revealed that inhibition of HDACs with Tucidinostat altered several critical pathways. Moreover, multiple epigenetic analyses suggested that Tucidinostat may alter the transcriptome via reprogramming the oncogenic epigenome and inducing the changes in microRNA-target interaction in uLMS cells. In the parallel study, we also determined the effect of DL-sulforaphane on the uLMS. Our study demonstrated the relevance of class I HDACs proteins in the pathogenesis of malignant uLMS. Further understanding the role and mechanism of HDACs in uLMS may provide a promising and novel strategy for treating patients with this aggressive uterine cancer.

## 1. Introduction

Uterine leiomyosarcoma (uLMS) is a rare and aggressive uterine cancer, representing 1–2% of all uterine malignancies [1]. The annual incidence of uLMS is approximately 0.8 per 100,000 women [2]. The five years survival for all patients is between 25 and 76%, with survival for women with metastatic disease at the initial diagnosis approaching only 10–15% [3]. Although irrespective of treatment, the uLMS is characterized by poor prognosis [4], the present treatment for uLMS patients exhibits resistance to currently available therapies, as evidenced by high recurrence and progression rates [5]. The origin and mechanism underlying driving its clinical and biological behavior remain unclear [6].

Histone deacetylases are a class of enzymes that remove acetyl groups from an ε-N-acetyl-lysine amino acid on histone and govern the chromatin structural dynamics of histones to wrap the DNA more tightly [7,8]. Its action is the opposite of histone acetyltransferase, which neutralizes their positive charge by acetylation of histone tails, thereby relaxing chromatin structure due to greater electrostatic repulsion from negatively charged DNA. Accumulated studies demonstrated that HDACs are involved in many biological events and pathological diseases [7,9,10,11], including varied types of cancer. Class I (HDACs 1, 2, 3, and 8) are located mainly in the nucleus and play an important role in cell proliferation, cell cycle progression, DNA damage response, development, and establishment and maintenance of the abnormal phenotype of diseases, including cancer progression [12,13,14,15,16]. HDACs 1, 2, and 3, as members of the class I HDAC family, are of particular interest as they are recruited to multiprotein complexes to mediate gene expression [17]. In addition, the modulation of these HDACs presents a specific possibility of interfering with multiple signaling pathways that are hijacked by tumor cells [12].

HDAC inhibitors (HDACi) have been used in several clinical studies and approved by the FDA for treating diseases and several types of cancer, including rare cancer [12,18,19,20,21]. Notably, treatments of tumor cells with HDAC inhibitors induce multiple effects, including cell cycle arrest, apoptosis, differentiation and senescence, modulation of immune response, altered angiogenesis, and restoration of sensitivity to drugs. The latter leads to the more promising outcomes, when the strategy of using combination treatments of HDACi with other chemotherapeutic agents is applied [22,23,24,25,26]. However, the role of HDACs in the pathogenesis of rare cancer, uLMS, is mainly unknown. In this study, we assessed the expression pattern of Class I HDACs in cells and tissues of uLMS and myometrium and characterized the role and mechanism of class I HDACs in the pathogenesis of uLMS. Deep diving into the molecular mechanism of uLMS pathogenesis linking to HDACs would help improve the clinical management and health outcomes of these discriminated patients.

## 2. Materials and Methods

### 2.1. Uterine Leiomyosarcoma Samples

The uLMS tissues were obtained from the University of Chicago Tissue Bank. Approval from the Institutional Review Board (# 20-1820) at the University of Chicago was obtained for the retrospective chart review of uLMS patients. Informed consent was obtained from all the study participants before surgery. The cases with an initial diagnosis of uLMS at the University of Chicago Hospital were reviewed, and the diagnosis was confirmed by hematoxylin-eosin (H&E) evaluation and immunohistochemistry. A total of nine cases with uterine uLMS were used as previously described [27].

### 2.2. Immunohistochemistry

Immunohistochemistry (IHC) was performed as described previously [27]. The primary antibodies used for IHC were shown in Table 1. To determine the percentage and intensity of HDACs-positive cells, QuPath software (version 0.2.3) (https://qupath.github.io, accessed on 27 October 2021) was used with the positive cell detection command. Thresholds were set to categorize cells according to nuclei staining intensity: negative, weak, moderate, and strong intensity. The histochemical scoring (H-score) captures both the intensity and the proportion of the HDAC-positive cells from the IHC image and comprises values between 0 and 300 [28], thereby offering a dynamic range to quantify HDACs abundance between myometrium and uLMS. Human testis, bladder, and colon tissues were used as positive tissues for HDAC1, HDAC2, and HDAC3, respectively.

### 2.3. Cells and Reagents

The source and culture condition of human leiomyoma cell line (HuLM), uterine smooth muscle (UTSM) cell line, SK-UT1 cell line (ATCC, Manassas, VA, USA), and MES-SA cell line (ATCC, Manassas, VA, USA) were described previously [27].

Class I HDAC inhibitor Tucidinostat was purchased from Selleck Chemical (Cat# S8567, Houston, TX, USA). DL-sulforaphane was purchased from Sigma-Aldrich (Cat# S4441, Saint Louis, MO, USA). The range of doses tested was 1–25 µM.

### 2.4. Proliferation Assay

A trypan blue exclusion assay was performed for Cell proliferation measurement. Cells were seeded into 12-well tissue culture plates and treated with the Tucidinostat and DL-sulforaphane at a dose range of 1–25 µM for 48 hr. An equal amount of DMSO was used as vehicle control. After treatment, the cells were trypsinized and collected by centrifuge. The cells were resuspended in a serum-free medium. Equal volume of 0.4% trypan blue and cell suspension was mixed and applied to a hemacytometer for cell counting. Viable cells were unstained. This assay was performed three times in triplicate.

### 2.5. Protein Extraction and Western Blot

Protein extraction and specific protein bands visualization were performed as described previously [27]. The information about usage of primary antibodies is listed in Table 1. The antigen-antibody complex was detected with Trident Femto Western HRP substrate (GeneTex, Irvine, CA, USA). Specific protein bands were visualized using ChemiDoc maging system (Bio-Rad, Hercules, CA, USA).

### 2.6. RNA-Sequencing

The uLMS cell line (SK-UT-1) was treated with 5 µM Tucidinostat or 5 µM DL-Sulforaphane for 48 hr. Cells were subjected to RNA isolated using Trizol. RNA and library quality and quantity were assessed as described previously [27]. An Illumina NovaSEQ6000 was used for library sequencing. 

### 2.7. Transcriptome Profiles Analysis

**Figure 1:**
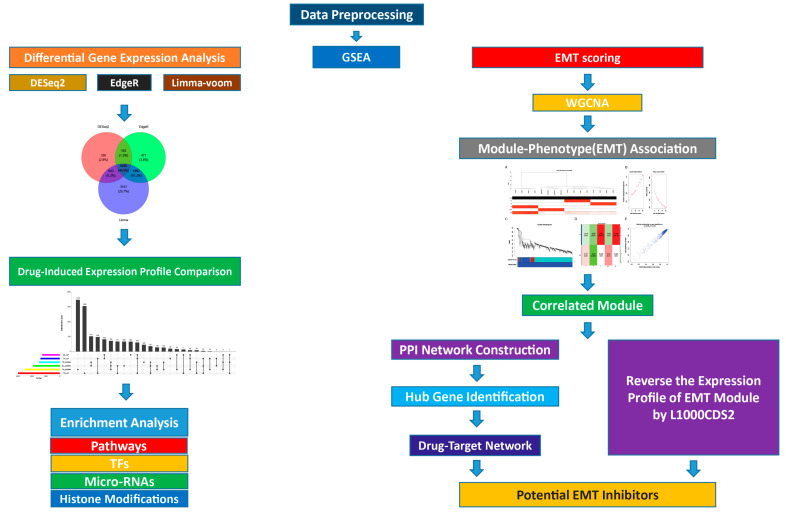
Flowchart of bioinformatics analysis.

We designed the bioinformatics analyses on the basis of the flow diagram, as shown in Figure 1.

#### 2.7.1. Transcriptome Data Analysis

The classical alignment-based mapper STAR, version 2.6.1d (GitHub, Inc., San Francisco, CA, USA) (23) was used to map sequencing reads to a human reference transcriptome. The results of STAR mapping were quantified by Salmon, version 1.4.0. Then, Bioconductor (https://bioconductor.org/packages/release/bioc/html/tximport.html, accessed on 27 October 2021) was used to read Salmon outputs into the R environment. Downstream analyses were performed as described previously [27].

#### 2.7.2. Differential Gene Expression Analysis

To identify the differentially expressed genes (DEGs) between treatment and control groups, three count-based algorithms were implemented in R packages DESeq2 [29], edgeR [30], and Limma + voom [31]. For each of these three methods, we used a cutoff −1.5 > fold-change > 1.5 and a *p*-value of 0.05. In addition, Benjamini and Hochberg’s (BH) method was performed to control the false discovery rate of all the genes with adjusted *P*-value less than 0.05.

#### 2.7.3. Gene List Enrichment Analysis

Comprehensive gene set enrichment analysis for regulation machinery was carried out using the enrichR (version 3.1) [32] package in R (https://maayanlab.cloud/Enrichr/ (accessed on 27 October 2021). We used ENCODE Histone Modifications 2015 for histone modification enrichment, ENCODE and ChEA Consensus TFs for transcription factor enrichment and TargetScan microRNA for microRNAs enrichment in EnrichR to determine the mechanisms underlying the regulation of DEGs.

#### 2.7.4. Drug Similarity Analysis

The L1000CDS2 is a pharmacogenetic search engine which enables users to find consensus L1000 small molecule signatures that match user input signatures. On the other hand, L1000CDS2 provides prioritization of thousands of small-molecule signatures, and their pairwise combinations. We performed drug similarity analysis using L1000CDS2 to investigate whether Tucidinostat and DL-sulforaphane induce the similar transcriptional changes with well-known HDAC inhibitors or not.

#### 2.7.5. Visualization of Aggregates and Intersection DEGs on UpSet Plot

The UpSet plot was constructed to explore the interactive gene sets among different drug-induced expression profiles using R package UpSetR (version 1.4.0) [33]. For up-regulated and down-regulated genes in all treatment, an eigengenes was calculated using the function module Eigengenes from WGCNA R package. Eigengenes is the vector that best describes the expression behavior of all genes within the module in the samples included in the analysis. To deep dive into pathways and relevant mechanisms of drugs, we integrated the present drugs (Tucidinostat and DL-sulforaphane) with our previous drug (TP472) to compare drug-induced expression profile.

#### 2.7.6. Epithelial–Mesenchymal Transition Score Calculation

We used 76GS [34], KS [35], and ssGSEA [36] methods to calculate the Epithelial–mesenchymal transition (EMT) scores for samples in the data set to investigate whether the drugs induced EMT or not.

#### 2.7.7. Co-Expression Network Analysis

The WGCNA R package was applied to construct co-expression network [37]. The top 10% most variable genes were selected for co-expression analysis. Cluster tree sampling was calculated using the flashCust function in R to find out and exclude outlier samples in which Z.K values were under −2.5. Scale-free topology criterion was used to choose the power value in which degree of independence fits 0.85. WGCNA has detected modules with high correlation, which a minimum number of genes in each module determined as 30, a cut height of 0.25, and a deep split level of 2.

Module membership (MM) and module-clinical trait relationship was calculated using a correlation between module eigengene (ME) -the best summary of module expression based on the first principal component- and the special phenotype (EMT score, etc.) that can lead to discover key biological functions (BP) and recognize associated key biomarkers. MM calculation was used to choose the module for further analysis.

The EnrichR web server (version 3.1) was used to perform pathway enrichment analyses (https://maayanlab.cloud/Enrichr/ (accessed on 27 October 2021)). First, STRING database (https://string-db.org/ (accessed on 27 October 2021)), which is the online search tool to protein–protein network (PPI) construction, was used to reconstruct the modules network (A combined score ≥ 0.4 of PPI pairs was considered significant), then the Cytoscape software [38] was employed to analyze and visualize the network.

### 2.8. Identification of the Potential Drugs

#### 2.8.1. Drug-Gene Interaction Network

The Drug Gene Interaction Database (DGIdb, v4.2.0) was used to identify potential drugs for EMT inhibition [39]. This database contains drug–gene interaction information from 22 source databases. To identify drug–gene interactions, only the approved interactions were considered.

#### 2.8.2. Module-Based Drug Prediction

As an alternative approach, we used L1000CDS2 to identify candidate drugs for inhibition of EMT module. With the L1000CDS2 tool, we prioritized small molecules that can potentially reverse gene expression [40]. In this study, the L1000CDS2 were applied to prioritize small molecules that are predicted to reverse the expression profile of the EMT module.

### 2.9. Statistical Analysis

A comparison of the two and multiple groups were carried out as described previously [27]. Data were presented as mean ± standard error (SE), and the significant difference was defined as *p* < 0.05.

## 3. Results

### 3.1. The Expression Levels of Class I HDACs Members Are Upregulated in uLMS Tissues Compared to Adjacent Myometrium from Women with uLMS

**Figure 2:**
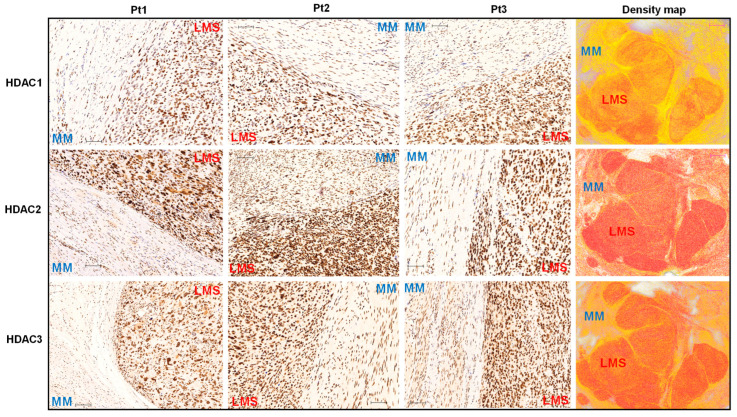
**IHC staining of HDAC1, 2, and 3 in human uLMS tissues and adjacent myometrium.** IHC staining for HDAC1, 2, and 3 is presented with three representative cases. The right column showed the density map of HDAC1, 2, and 3 for the same representative case. Blue color: negative; Yellow color: low expression; brown color: moderate expression; red color: strong expression. Scale bars in black color: 100 µm; Scale bars in red color: 1mm.

**Figure 3:**
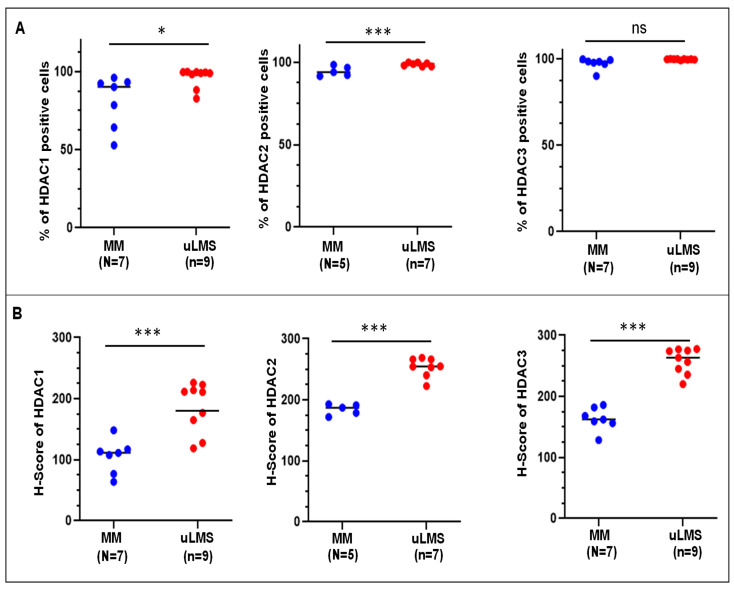
**Percentage of HDACs positive cells and H-score of HDACs expression in uLMS vs. myometrium.** (**A**) Percentage of HDAC1, HDAC2, and HDAC3 positive cells in uLMS and myometrium tissues; (**B**) H-score of HDAC1, HDAC2, and HDAC3 in uLMS and myometrium tissues. * *p* < 0.05. *** *p* < 0.001. ns: no significant difference.

To determine the differential expression levels of Class I HDAC proteins between uLMS (*n* = 9) and MM^+LMS^(*n* = 7), IHC staining for HDAC1, 2, and 3 was performed. We examined the IHC images of three HDAC proteins in uLMS tissues vs. myometrium (MM). Figure 2 and Figure 3 showed that the HDAC1 and HDAC2 positive cells were significantly higher in uLMS compared to MM^+LMS^. The H-score of HDAC1 and HDAC2 was also significantly increased in uLMS (*n* = 9) compared to MM (*n* = 7). Although HDAC3- positive cells showed no significant difference between uLMS vs. MM^+LMS^, the H-score of HDAC3 was significantly increased in uLMS compared to MM (Figure 2 and Figure 3), indicating the critical role of class I HDACs in the pathogenesis and progression of uLMS. Figure 2 (right column) revealed an increase in expression density of HDAC1, 2, and 3 in uLMS compared to MM^+LMS^.

### 3.2. Class I HDAC Protein Levels Are Upregulated in uLMS Cell Lines

**Figure 4:**
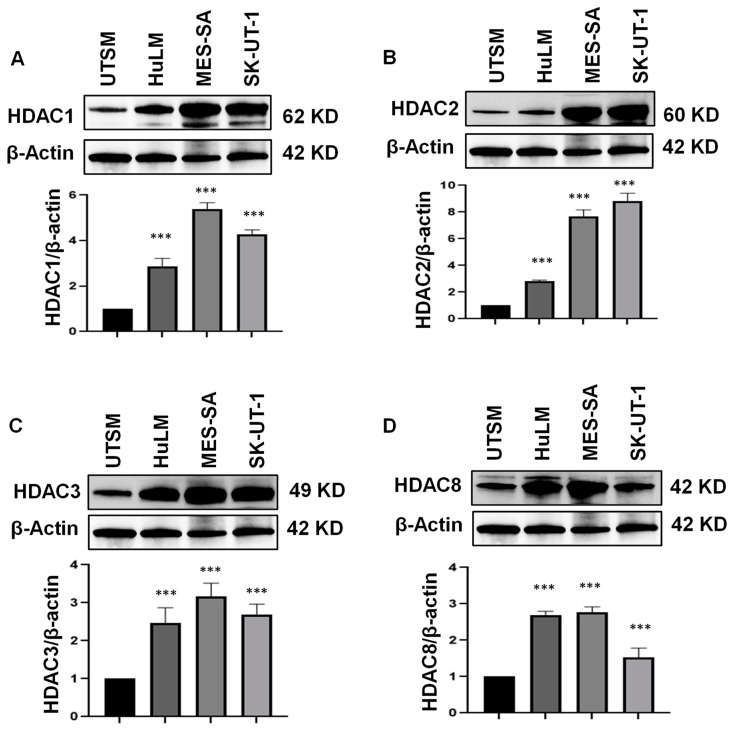
**The expression of Class I HDACs in UTSM, HuLM, MES-SA, and SK-UT-1 cell lines**. The protein levels of Class I HDACs (HDAC1 [(**A**), 2 (**B**), 3 (**C**), and 8 (**D**)] were measured by Western blot. β-actin was used as an endogenous control. Quantitative analysis of relative levels of HDAC1, 2, 3, and 8 (**A**–**D**) was performed using Image J (1.53t version) (NIH, Bethesda, MD, USA). *** *p* < 0.001.

The constitutive (basal) expression levels of Class I HDAC components in UTSM, HuLM, and uLMS cell lines were evaluated by immunoblot analysis. We demonstrated that the protein levels of HDAC1, 2, and 3 exhibited a graded increase from normal and benign UF tumor cells to malignant uLMS cells (*p* < 0.05) (Figure 4A–C). In addition, although the protein levels of HDAC8 were not increased in uLMS compared to HuLM cells, a significant upregulation of HDAC8 was observed in two uLMS cell lines compared to the UTSM cell line (*p* < 0.05) (Figure 4D).

### 3.3. Inhibition of HDACs Decreased the Cell Proliferation in uLMS Cells

**Figure 5:**
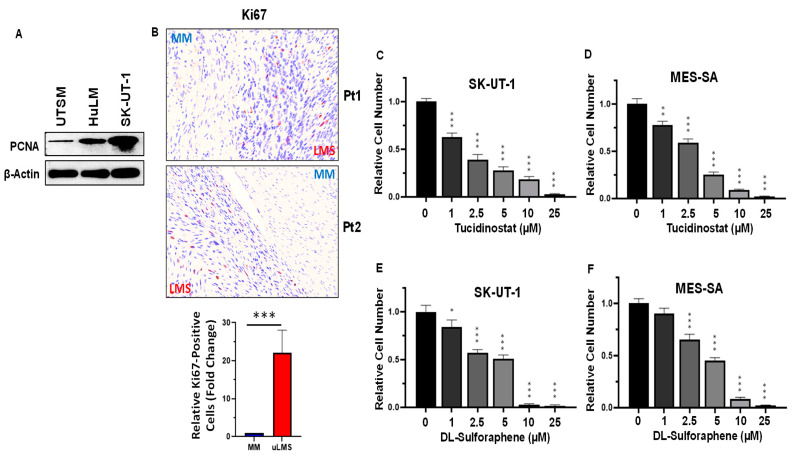
**Treatment with Tucidinostat and DL-sulforaphane decreases uLMS cell proliferation**. (**A**): Abnormal cell proliferation in uLMS cells validated by Western blot using anti-PCNA antibody; (**B**): IHC staining revealed the increased Ki67-positive cells in uLMS tissues compared to the myometrium. Lower panel is the quantitative analysis of Ki67-positive cells between uLMS and adjacent myometrium (*n* = 3) using QuPath software. (**C**): Cell proliferation in SK-UT-1 cells in the presence or absence of Tucidinostat; (**D**): Cell proliferation in MES-SA cell line in the presence or absence of Tucidinostat; (**E**): Cell proliferation in SK-UT-1 cell line in the presence or absence of DL-Sulforaphane; (**F**): Cell proliferation in MES-SA cell line in the presence or absence of DL-Sulforaphane.* *p* < 0.05; ** *p* < 0.01; *** *p* < 0.001.

Abnormal cell proliferation is common in many types of cancer. We detected the proliferation in UTSM, HuLM, and SK-UT-1 cell lines by Western blot using the antibody against PCNA, the cell proliferation marker. As shown in Figure 5A, the levels of PCNA in SK-UT-1 were highest among the three cell lines, suggesting that the uLMS cell line SK-UT-1 grew fastest. In addition, the PCNA levels in HuLM cell line are higher than in UTSM cell lines. In addition, we detected the Ki67-positive cells in uLMS and adjacent myometrium tissues. As shown in Figure 5B, a significant increase in Ki67-positive cells was observed in uLMS tissues compared to myometrium tissues.

Tucidinostat (chidamide), as a Class I HDAC inhibitor, has been shown to inhibit a variety of cancer growth [41,42,43,44,45,46]. Therefore, we selected Tucidinostat in our in vitro cell model to assess its effect on uLMS cell growth. The trypan blue exclusion assay was performed in SK-UT-1 and MES-SA cell lines treated with dose ranges from 1–25 µM. Treatment with Class I HDAC inhibitor (Tucidinostat) for 48 h showed a dose-dependent inhibitory effect on the proliferation of both SK-UT-1 and MES-SA cells (Figure 5C,D).

Sulforaphane is an isothiocyanate present naturally in widely consumed vegetables and has been shown to have an inhibitory effect on various cancers [47,48,49,50,51,52,53]. In addition, DL-sulforaphane occurs naturally as L-isomer in edible cruciferous vegetables such as broccoli [54,55]. This study tested the effect of DL-sulforaphane on uLMS cell growth. Similarly, treatment of uLMS cell lines with DL-sulforaphane exhibited a dose-dependent cell growth inhibition for 48 h (Figure 5E,F). Therefore, both Tucidinostat and DL-sulforaphane elicit the anti-proliferation effect on uLMS cells.

### 3.4. Tucidinostat and DL-Sulforaphane Sculpt the Transcriptome of uLMS Cells

**Figure 6:**
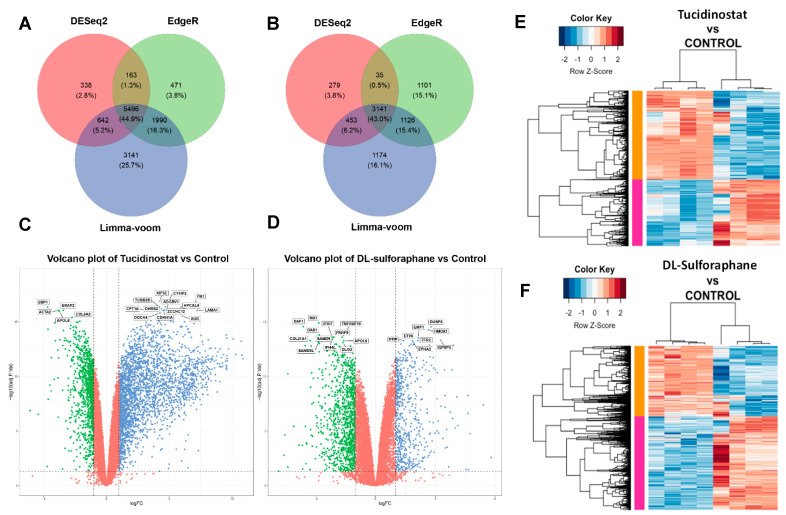
**Treatment with Tucidinostat and DL-sulforaphane sculpts the transcriptome of uLMS cells.** Venn diagrams to demonstrate the DEGs identified by three methods of Limma + voom, edgeR and DESeq2 at Adjusted *P*-value cut off 0.05 and −1.5 > log2FC > 1.5 for (**A**) Tucidinostat vs. Control (**B**) DL-sulforaphane vs. Control. Volcano plots of the gene expression profiles of (**C**) Tucidinostat vs. Control (**D**) DL-sulforaphane vs. Control. The blue points represent upregulated genes, and the green points represent downregulated genes. The vertical dotted black lines represent the log (FC) cutoff, and the horizontal black line represents the logarithmic transformed Adjusted *P*-value cutoff. (**E**) Heat map; Pearson correlation was used to cluster DEG (Tucidinostat vs. Control), which were then represented as a heatmap with the data scaled by Z score for each row. (**F**); Heat map. Pearson correlation was used to cluster DEG (DL-Sulforaphane vs. Control), which was then represented as a heatmap with the data scaled by Z score for each row. DEGs: differentially expressed genes, FC: fold-change.

To characterize the Tucidinostat and DL-sulforaphane-induced transcriptional changes in uLMS cells, RNA-sequencing analysis was performed in control (DMSO, *n* = 4), Tucidinostat-treated uLMS cells (*n* = 4), and DL-sulforaphane-treated uLMS cells (*n* = 4). Tucidinostat yielded 6639 DEGs (2846 down, 3793 up), and DL-sulforaphane yielded 3908 DEGs (2226 down, 1682 up). Tucidinostat and DL-sulforaphane upregulated 28% and 12.4% of gene expression and downregulated 21% and 16.4% of gene expression. Differential gene expression analysis was done by three algorithms: limma-voom, DESeq2, and edgeR. The highest number of DEGs between Tucidinostat and control samples (11,269) and DL and control samples (5894) was identified using Limma + voom. EdgeR identified 8120 and 5403 DEGs, respectively (Figure 6A,B). DESeq2, on the other hand, identified the lowest number of DEGs (6639 and 3908). A set of 5496 and 3141 genes was common in the three differential gene expression analysis methods (Figure 6A,B). Figure 6C,D revealed the distribution of DEGs between treatments and DMSO control. Figure 6E,F exhibited distinct expression patterns between DMSO control vs. Tucidinostat and DL-sulforaphane, respectively.

#### 3.4.1. Pathway Analysis of DEGs upon Tucidinostat and DL-Sulforaphane Treatment

**Figure 7:**
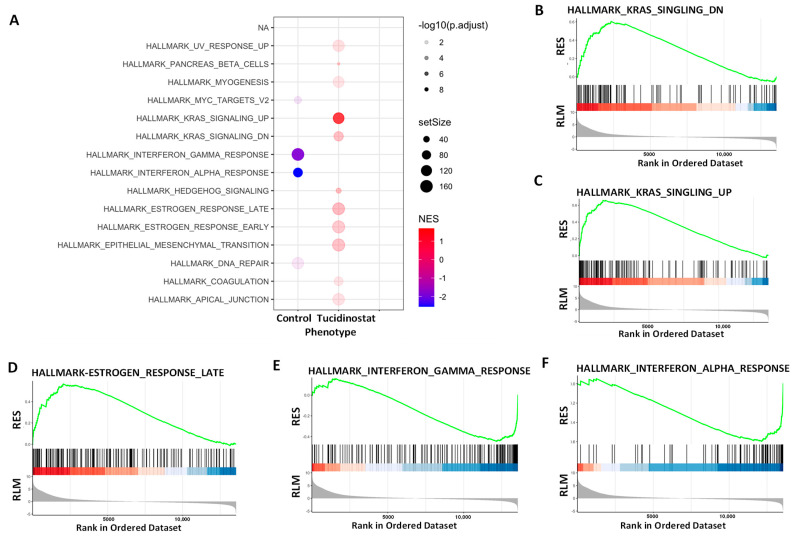
**Hallmark analysis demonstrated the alteration of multiple pathways in SK₋UT₋1 cells in response to Tucidinostat (TD) treatment (Significantly enriched gene sets (HDAC inhibitor vs. control) from GSEA using Hallmark biological processes in MSigDB**. (**A**) Functional pathways analysis identified significantly altered pathways in SK₋UT₋1 cells treated with Tucidinostat. Significantly enriched gene sets (Tucidinostat vs. control) from GSEA using Hallmark biological processes in MSigDB. Gene count and significance levels are shown by the size and color of each circle, respectively. Pathways analysis revealed that several gene sets were altered and associated with KRAS signaling (**B**,**C**), estrogen-response (**D**), interferon-response (**E**,**F**). RES: Running Enrichment Score; RLM: Ranked list metric.

To gain insight into the biological changes by HDAC inhibition, gene set enrichment analysis (GSEA) was performed. We demonstrated that several gene sets were enriched in Tucidinostat vs. control group (Figure 7A–F).

**Figure 8:**
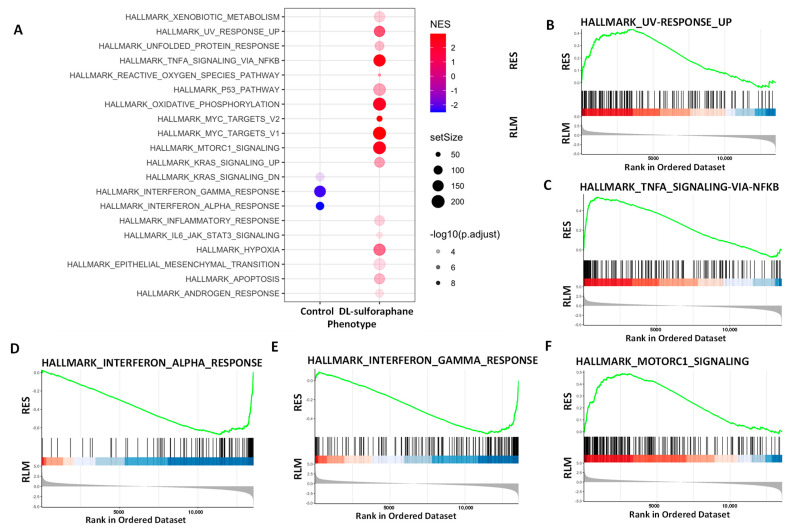
**Hallmark analysis demonstrated the alteration of multiple pathways in SK₋UT₋1 cells in response to DL-sulforaphane treatment (Significantly enriched gene sets (DL-sulforaphane vs. control) from GSEA using Hallmark biological processes in MSigDB**. (**A**) Functional pathways analysis identified significantly altered pathways in SK₋UT₋1 cells treated with DL-sulforaphane t. Significantly enriched gene sets (DL-sulforaphane vs. control) from GSEA using Hallmark biological processes in MSigDB. Gene count and significance levels are shown by the size and color of each circle, respectively. Pathways analysis revealed that several gene sets associated with UV response (**B**), TNFa via NFkB (**C**), Interferon-alpha (**D**), Interferon-gamma (**E**), and MTORC1 (**F**) were altered. RES: Running Enrichment Score; RLM: Ranked list metric.

GSEA analysis also revealed that DL-sulforaphane altered the expression of several gene sets (Figure 8A), including UV response (Figure 8B), TNFa signaling via NFkB (Figure 8C), interferon alpha/Gamma response (Figure 8D,E), MTORC1(Figure 8F), xenobiotic metabolism, reactive oxygen species pathway, P53 pathway, oxidative phosphorylation, MYC targets, KRAS, among others. Notably, Tucidinostat and DL-sulforaphane shared altered common pathways, including UV response, MYC targets, KRAS signaling, interferon alpha/Gamma response, and inflammatory response (Figure 7A and Figure 8A).

#### 3.4.2. The Expression of Cell Cycle- and Apoptosis-Related Genes Is Altered upon Tucidinostat and DL-Sulforaphane Treatment

**Figure 9:**
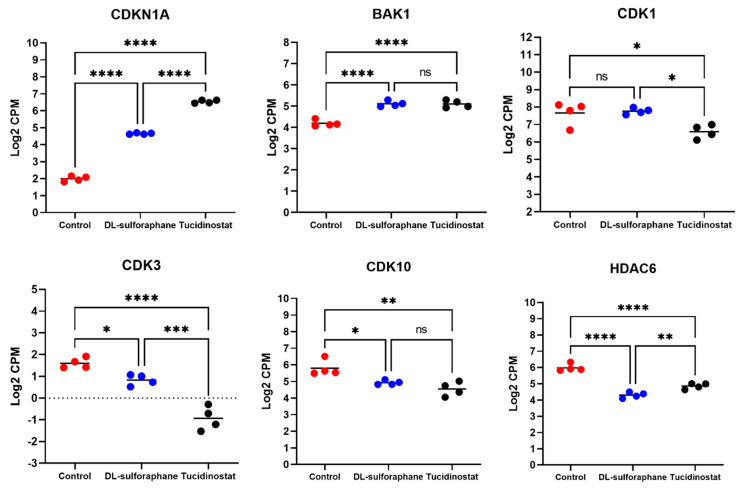
**Tucidinostat and DL**−**sulforaphane altered cell cycle- and apoptosis**−**related gene expression in uLMS cells.** RNA−seq revealed the upregulation of P21 and BAK1 and downregulation of CDK1, CDK3, CDK10, and HDAC6 in uLMS cells. NS: no significant difference; * *p* < 0.05; ** *p* < 0.01; *** *p* < 0.001; **** *p* < 0.0001.

To determine genes regulating the cell cycle and apoptosis in uLMS cells in response to Tucidinostat and DL-sulforaphane treatments, we checked the expression of *CDKN1A* (*P21*), *BAK1*, *CDK1*, *CDK3*, *CDK10*, and *HDAC6*. As shown in Figure 9, both Tucidinostat and DL-sulforaphane increased the expression of *CDKN1A* and *BAK1* and reduced the expression of *CDK3* and *CDK10*. In addition, Tucidinostat but not DL-sulforaphane reduced the expression of *CDK1*. Moreover, both Tucidinostat and DL-sulforaphane decreased the expression of HDAC6 in uLMS cells. BAK localizes to mitochondria, and functions to induce apoptosis. HDAC6 is involved in cell cycle and apoptosis [56], and *CDKN1A* and *CDK* members were critical in cell cycle progression [57,58,59]. Therefore, our results suggested that Tucidinostat and DL-sulforaphane suppressed the uLMS proliferation via cell cycle arrest and apoptosis.

HDACs have been implicated as a target for sulforaphane. A number of studies have demonstrated that sulforaphane impacts the cellular acetylome and decreases HDAC activity in several models and diseases, including prostate epithelial cells, mouse colon tissues, satellite cells, and human peripheral blood mononuclear cells [60,61,62], concomitantly with an increase in the levels of acetylated histones and p21. In addition, separate studies demonstrated that other types of cells, such as HCT116 is at least partially resistant to the nuclear HDAC inhibitory effect of Sulforaphane [63]. Therefore, sulforaphane exerted a distinct action on transcriptome changes in a cell-dependent manner.

To assess if Tucidinostat and DL-sulforaphane exhibited similar transcriptional pattern in uLMS with other HDAC inhibitors, we performed drug similarity analysis using L1000CDS2 and demonstrated that Tucidinostat- induced transcriptional pattern has similarity with signatures of multiple HDAC inhibitors, including BRD-K11663430, HDAC6 inhibitor ISOX, mocetinostat, vorinostat, entinostat, among others, which were shown in Table S1. However, the transcriptional signature induced by DL-sulforaphane did not show similarity with any other HDACi-induced transcriptional pattern (Table S2), indicating that DL-sulforaphane may not target HDAC directly in uLMS cells.

#### 3.4.3. Tucidinostat and DL-Sulforaphane Altered the Gene Expression Correlating to Histone Modifications

**Figure 10:**
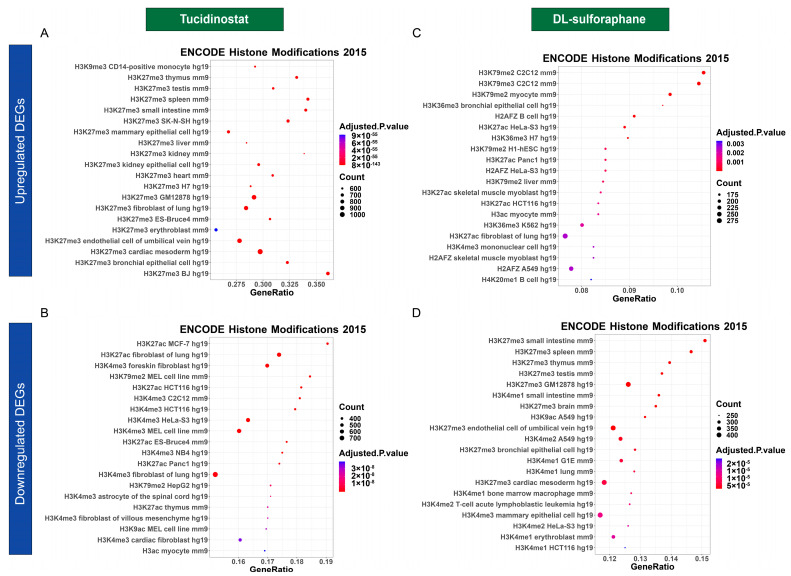
**Dot plot analysis for histone modifications**. The dot plots showed the top twenty enrichment terms for histone modification associated with up DEGs (**A**) and down DEGs (**B**) in response to Tucidinostat treatment. (**C**) histone modifications related to up DEGs in response to DL-sulforaphane treatment. (**D**) histone modifications associated with down DEGs in response to DL-sulforaphane treatment. The *X*-axis represents the gene ratio, and the *y*-axis descript the enrichment components. The area of the circle is proportional to the number of genes assigned to the term, and the color accords with the Adjusted *p*-value.

To understand epigenetic-mediated transcriptional changes in response to the Tucidinostat and DL-sulforaphane treatment, we performed enrichment analysis of epigenetic histone markers using the Enrichr web server. As shown in Figure 10, several histone modifications, including H3K27me3 and H3K9me3, associated with upregulated DEGs in response to Tucidinostat were identified (Figure 10A). In addition, down-regulated DEGs associated with histone modifications, including H3K27ac, H3K4me3, and H3K79me2 were identified (Figure 10B). For DL-sulforaphane-induced DEGs associated histone modifications, our analysis revealed that histone modifications with up DEGs included H3K27me3, H3K27me2, H3K27ac, H3K79me2, among others (Figure 10C). The histone modifications associated with DL-sulforaphane-induced down DEGs included H3K27me3, H3K4me1, H3K9ac, H3K4me2, among others (Figure 10D)

#### 3.4.4. Tucidinostat and DL-Sulforaphane Altered the Gene Expression Associated with Transcriptional Factors

**Figure 11:**
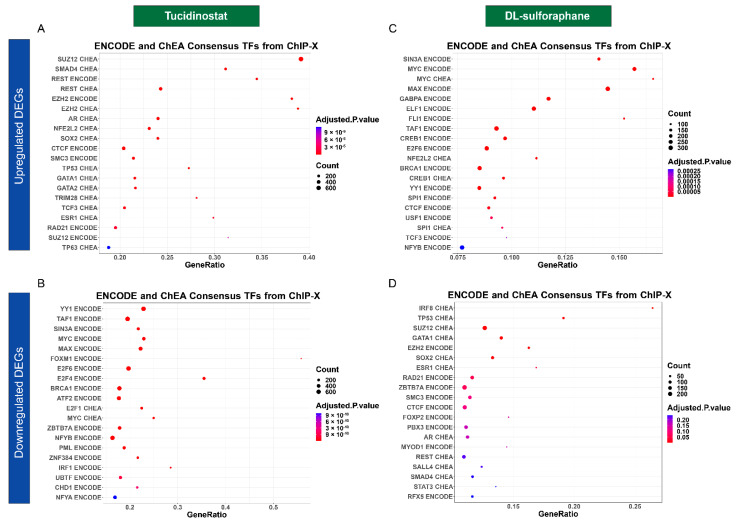
**Transcription factors enrichment analysis**. The dot plots showed the top twenty enrichment Terms for transcription factors associated with up DEGs (**A**) and down DEGs (**B**) in response to Tucidinostat treatment. (**C**) Transcriptional factors associated with up DEGs in response to DL-sulforaphane treatment. (**D**) Transcriptional factor enrichments associated with down DEGs in response to DL-sulforaphane treatment.

Transcriptional factors play an important in many biological processes and their control is disrupted in cancer cells [64]. The dysregulation of these core transcription factors forms interconnected transcriptional loops to establish and reinforce the abnormal gene-expression program in cancer cells [65]. Our studies demonstrated that Tucidinostat and DL-sulforaphane induced- DEGs are putative targets of multiple transcriptional factors, as shown in Figure 11A–D.

#### 3.4.5. Tucidinostat and DL-Sulforaphane Altered the Gene Expression Correlating to the miRNA Regulation

**Figure 12:**
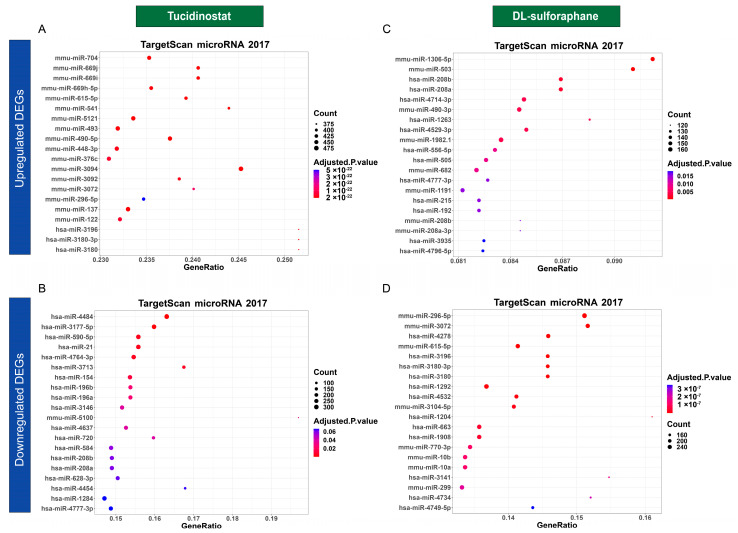
**MicroRNAs enrichment analysis.** The dot plots showed the top twenty enrichment Terms for microRNAs associated with up DEGs (**A**) and down DEGs (**B**) in response to Tucidinostat treatment. (**C**) MicroRNAs associated with up DEGs in response to DL-sulforaphane treatment. (**D**) MicroRNAs associated with down DEGs in response to DL-sulforaphane treatment.

We used TargetScan microRNA analysis in Enrichr web server to determine the mechanism underlying the regulation of DEGs associated with miRNAs in response to HDAC inhibitors treatment. As shown in Figure 12A–D, Tucidinostat and DL-sulforaphane induced- DEGs are putative targets of multiple miRNAs.

#### 3.4.6. UpSet Plot Visualization

**Figure 13:**
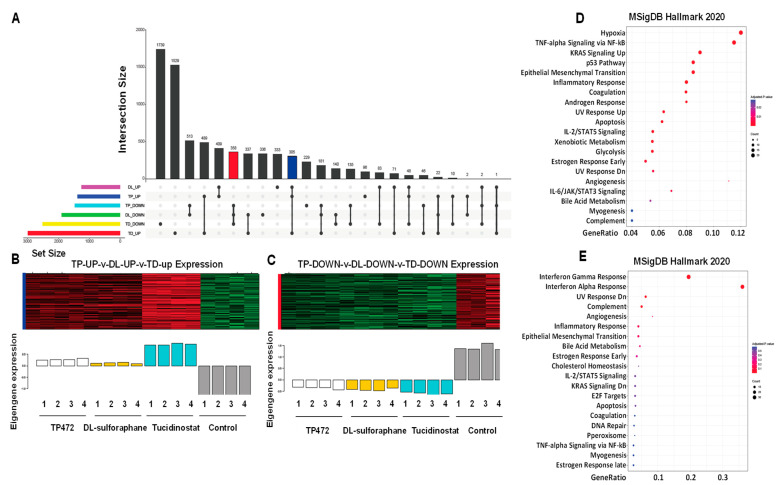
**Intersection size of DEGs across Tucidinostat, DL**−**sulforaphane, and TP472 treatments and associated epigenetic changes**. (**A**) Upset diagram showing the intersection size of upregulated and downregulated genes across drug treatments. (**B**) Eigengenes expression pattern in the blue intersection. (**C**) Eigengenes expression pattern in the red intersection. (**D**,**E**) The dot plot of the top 20 functional enrichment results for (**D**) blue intersection and (**E**) red intersection.

To further visualize the intersections of three drugs-induced DEGs (Tucidinostat up, Tucidinostat down, DL-sulforaphane up, DL-sulforaphane down, TP472 up, TP472 down), the upset plot was utilized to present the distribution characteristics of DEGs upon treatments. As shown in Figure 13A, the common up and down DEGs with Tucidinostat and DL-sulforaphane contain 71 and 140 genes, respectively. The TP472-down/DL-sulforaphane-down groups had 513 genes, the group with the largest number of genes in all groups with genes involved in two types of gene regulations. In addition, the Tucidinostat-down/DL-sulforaphane-down/TP472-down groups contained 358 genes, the group with the largest number of genes in all groups involved in three types of regulations. Figure 13B,C exhibited the expression pattern in the red intersection (Tucidinostat-down, DL-sulforaphane-down, and TP472-down), the blue intersection (Tucidinostat-up, DL-sulforaphane-up, and TP472-up) presented in Figure 13A.

Functional enrichment analysis revealed that the genes from the blue and red intersections in Figure 13A exhibited multiple enriched pathways, including inflammatory response, p53 pathway, KRAS signaling, apoptosis, estrogen response, UV response, complement, angiogenesis, IL-2/STAT5 signaling, EMT, among others (Figure 13D,E).

Since the number of shared DEGs between Tucidinostat and DL-sulforaphane are much less compared to the number of DEGs from each individual group, one may consider that Tucidinostat and DL-sulforaphane promoted the inhibitory effect on uLMS via different network mechanisms.

#### 3.4.7. Apoptosis and EMT

**Figure 14:**
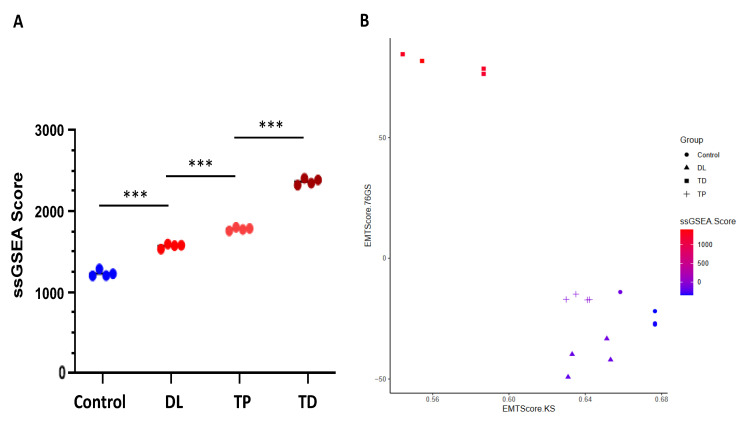
**Apoptosis and EMT scoring**. The *X*-axis represents the 76 gene signature scores, and the *y*-axis describes the Kolmogorov–Smirnov test scores. (**A**) ssGSEA scoring of hallmark apoptosis pathway, (**B**) EMT scoring: Dot shape indicates group, and dot color indicates ssGSEA score for EMT. ssGSEA: Single-sample gene set enrichment analysis. *** *p* < 0.001, DL: Dl-sulforaphane; TP: TP472; TD: Tucidinostat.

To further determine the apoptosis pathway affected by the treatments, we used the single-sample gene set enrichment analysis (ssGSEA) to measure the apoptosis scoring in response to the Tucidinostat, DL-sulforaphane, and TP472. We demonstrated that treatments with above three agents significantly increased the apoptosis scoring compared to the control (DMSO) group. Moreover, Tucidinostat exhibited the most effective of activating apoptosis among the three drugs (Figure 14A).

For epithelial-mesenchymal transition (EMT) scoring, we used three different methods to quantify EMT; 76GS, KS, and ssGSEA. The KS method has a predefined scale for EMT scores [−1, 1], with higher scores indicating mesenchymal signatures. On the other hand, there is no pre-defined range of scores calculated by 76GS, and the higher the 76GS score, the more epithelial the sample is. The score derived from ssGSEA reflects the degree to which the input gene signature is coordinately up or downregulated within samples (Figure 14A,B). 

#### 3.4.8. Co-Expression Network Analysis

**Figure 15:**
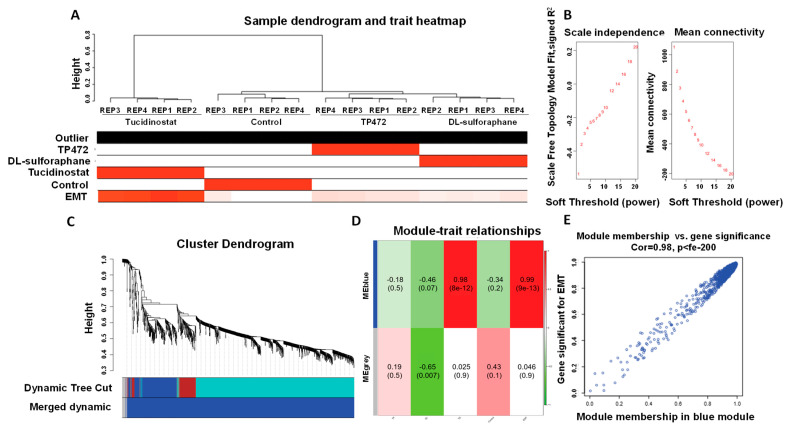
**Sample dendrogram and trait heatmap.** (**A**) The five traits are Tucidinostat, DL−sulforaphane, TP−472, Control, and EMT. (**B**) Scale independence and mean connectivity of various soft-thresholding values. (**C**) Gene co-expression network modules. Different colors represent different modules, and gray represents genes that cannot be merged into any module. The bottom colors represent the module after merging modules. (**D**) Heatmap of the correlation between the clinical traits and MEs. Each cell contains the corresponding correlation and the *P* value. The table is color-coded by the correlation according to the color legend. (**E**) Using linear regression, scatter plots of gene significance (GS) vs. module membership (MM) in the blue modules.

**Figure 16:**
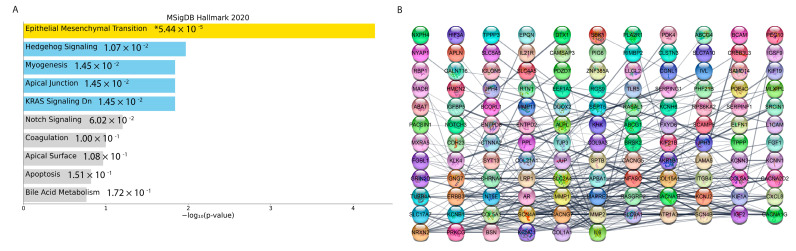
**Protein**−**protein interaction analysis and model**. (**A**) Bar plot for pathway enrichment of genes in the blue module. (**B**) Protein−protein interactions of top genes in the opposite module in a grid layout sorted by degree. * *p* < 0.05.

The weighted gene co-expression network was constructed with 1356 most variable genes (the top 10% of genes). (Figure 15A) The scale-free topology R2 did not reach the soft threshold of 0.85, so the recommended power value of 12 was chosen (Figure 14B). The WGCNA revealed two gene co-expression modules by average linkage hierarchical clustering. Modules with more than 0.25 expression profiles similarity were merged. Each module was shown in a unique color (Figure 15C). The Module-Blue was found to have the highest significant association with EMT (correlation = 0.99 and *p*-value < 9 × 10^−13^). (Figure 15D,E). The 255 genes were found in the Module-Blue, which used the STRING tool to reconstruct the gene-gene interactions (https://string-db.org/ (accessed on 27 October 2021). Cytoscape software identified the hub genes associated with the Module-Blue. Based on intramodular connectivity, top hub genes in the co-expression network are shown in Figure 16.

### 3.5. Potential Drugs Prediction

This study used DGIdb as the first approach for identifying the possible drugs with the effects on reversing the increased expression of EMT hub genes. Using DGIdb, we found 186 candidate drugs that target the top 10 percent of EMT modules’ genes (based on the network’s connectivity). These potential drugs are shown in Table S3.

Our research used L1000CDS2 as the second approach to identify drug candidates for EMT inhibition. The list of the EMT modules DEGs and their related fold changes between the Tucidinostat group (as the most significant treatments group) vs. Control was entered to the L1000CDS2 web tool to search for molecular compounds that can reverse the expression changes. The top 50 drugs are identified as an output and are shown in Table S4. This study proposes future research to use a combination treatment strategy (HDACi in combination with EMT inhibitors) to achieve a better outcome in treating human uLMS.

## 4. Discussion

ULMS is a highly aggressive tumor with high tumor recurrence rates, progression, and metastasis [5]. The malignant potential of uterine fibroids is extremely low [66,67]. The origin and molecular mechanism underlying driving its clinical and biological behavior remain unclear [6].

This study demonstrated that Class I HDACs are abnormally upregulated in uLMS compared to myometrial tissues, which may contribute to the uLMS pathogenesis.

We compared the expression levels of main class I HDAC members HDAC1, 2, and 3 in uLMS and adjacent myometrium, as well as the expression of full members of Class I HDACs in UTSM, HuLM, and uLMS cell lines. The protein levels of HDAC1, 2, and 3 are significantly upregulated in two uLMS cell lines compared to UTSM and HuLM cell lines. In addition, the expression levels of HDAC 1,2 and 3 are upregulated in uLMS tumors compared to myometrium tissues suggesting that Class I HDACs may contribute to the pathogenesis of uLMS.

Abnormal cell proliferation via decreasing the cell cycle arrest and apoptosis is common in many cancers [68,69,70,71]. We revealed that uLMS cells grow faster than myometrial cells in vitro and in vivo. To determine if targeting HDACs can suppress the uLMS phenotype, we determined the uLMS cell growth in response to HDAC inhibitor treatment. Our study demonstrated that HDAC inhibition suppressed the uLMS cell proliferation. The anti-tumor effect of HDAC inhibition observed in our model was consistent with literature from other types of cancer. Targeting class I HDACs with Tucidinostat (benzamide HDAC inhibitor) has been studied in different types of cancers showing beneficial effects. For instance, in pancreatic cancer, Tucidinostat treatment synergistically enhances gemcitabine cytotoxicity in pancreatic cancer cells [72]. In multiple myeloma (NN), Tucidinostat inhibited the proliferation and invasion of MM cells. In addition, Tucidinostat in combination with lenalidomide and a low dose of borezomid exhibited a synergistic effect in MM [73]. In hepatic cancer, Tucidinostat showed an anti-tumor activity [74]. Moreover, Tucidinostat targeted stem and progenitor cells of acute myeloid leukemia [75]. All those studies demonstrated the critical role of Class I HDACs in cancer development and targeting class I HDACs showed beneficial effects in several types of neoplasms.

To further determine the mechanisms associated with inhibition, we performed a genome-wide RNA-sequencing experiment comparing the DMSO-treated with Tucidinostat -treated uLMS cells. In addition, DL-sulforaphane-induced transcriptional changes were also compared. The transcriptome analysis revealed that targeting HDACs with Tucidinostat altered several critical biological pathways that may contribute to uLMS pathogenesis. In addition, we determined the transcriptional changes in response to DL-sulforaphane treatment. Although DL-sulforaphane showed the activity of inhibiting cell proliferation, the specificity of the drug targeting class I HDACs has not been reported in uLMS. Our analysis demonstrated that 21.5% and 27.5% of down DEGs shared common genes between Tucidinostat and DL-sulforaphane treatment groups. Similarly, 18.1% and 40.8% of up DEGs showed common genes between Tucidinostat and DL-sulforaphane treatment groups. This overlapped analysis is consistent with the hallmark analysis demonstrating that Tucidinostat and DL-sulforaphane impaired common pathways. However, the alteration of some other biological pathways differed in response to Tucidinostat and DL-Sulforaphane, respectively.

In contrast to Tucidinostat, the inhibitory effect of Sulforaphane on HDAC activity is controversial. For example, sulforaphane treatment does not reduce nuclear HDAC activity, but decreases the levels of HDAC1-4 and 6 in keratinocytes [63]. However, other studies demonstrated the inhibitory effect of sulforaphane on HDAC activity. For example, sulforaphane repressed the HDAC activity by 40%, 30%, and 40% in BPH-1, LnCaP, and PC-3 prostate epithelial cells, respectively. The HDAC inhibition was accompanied by a 50–100% increase in acetylated histones in all three prostate cell lines [61]. A similar study showed that sulforaphane dramatically reduced HDAC activity in porcine satellite cells with an increase in global acetylated H3 and H4 levels [62]. The in vivo studies demonstrated that sulforaphane could decrease HDAC activity by ~65%, concomitantly with an increase in acetylated histones globally, as well as locally on the promoters of genes such as P21 and BAX [60]. Therefore, sulforaphane may have a distinct impact on different cell types. In this study, our drug similarity analysis demonstrated that Tucidinostat-induced transcriptional signature had a similar pattern with several other known HDAC inhibitors-induced patterns. Still, DL-sulforaphane did not, indicating that DL-sulforaphane may affect uLMS via different mechanisms, rather than HDAC inhibition in uLMS. Notably, we demonstrated that the more inhibitory efficiency of Tucidinostat over DL-sulforaphane is consistent with the expression levels of cell cycle-related genes, including P21, CDK1, and CDK3, as shown in Figure 9. For the apoptosis-related gene, both Tucidinostat and DL-sulforaphane increased the expression levels of BAK1, which localizes to mitochondria, and functions to induce apoptosis. Moreover, Tucidinostat probably promoted apoptosis more strongly than DL-sulforaphane identified by GSEA Score. Therefore, targeting class I HDACs with Tucidinostat proves superior to DL-sulforaphane by inhibiting the uLMS proliferation via inducing apoptosis and cell cycle arrest in uLMS cells.

EMT is a cell biological process crucial for tumor aggressiveness, including cancer metastasis and drug response [76,77]. Therefore, the EMT status of cancer cells can be proved to be a critical estimate of patient prognosis. In this regard, we used three distinct metrics that score EMT on a continuum, based on the transcription signature of Tucidinostat and DL-sulforaphane and control groups. However, our results demonstrated that both drugs increase the EMT levels compared to the DMSO control (Figure 13B). It has been reported that dysregulation of apoptosis and EMT are linked with various pathological progress, including tumor formation and progression [78]. Notably, TGF-β, as a potent pleiotropic molecule, induced apoptosis and simultaneously induced the EMT of AML-12 cells. The question is if targeting the HDACs induces these concurrent but distinct events in uLMS cells. A study by Yang et al. demonstrated that TGF-β1-induced apoptosis and EMT were closely related to the cell cycle stage, and TGF β 1-induced concurrent apoptosis and EMT are independent of each other [79]. Therefore, deep diving into the mechanism underlying the Tucidinostat and DL-sulforaphane-induced changes in apoptosis, EMT, and other pathways is worthwhile.

It has been reported that cross-talk between different epigenetic mechanisms regulates gene expression [80,81,82,83]. For instance, HDAC inhibitors can elicit transgenerational effects via altered DNA and histone methylation [84]. HDAC4 and HDAC9 can differentially influence H3K27 acetylation, which can explain the pleiotropic actions of MEF2 transcription factors in uLMS [85]. In this study, Tucidinostat altered the up-DEGs correlated with multiple histone modifications in uLMS, including H3K27me3, H3K79me2, 3, and H3K9ac, among others. The down-DEGs correlated with H3K27ac, H3K4me1, 2, 3, and H3K27me3, among others. Our studies indicated that HDAC inhibition might alter the transcription by reprogramming the oncogenic transcriptome in uLMS. This observation was further confirmed by transcriptional factor regulation analysis inferred from integrating genome-wide ChIP-X (ChIP-chip, ChIP-seq, ChIP-PET, and DamID) studies. By combining transcriptome data with ChIP-X transcriptional factor, and histone modification studies, we identified multiple putative networks linking to enriched pathways, that can help target specific transcription factor activity in uLMS cells using combination drug treatment strategy.

Notably, the interplay between miRNAs and HDACs has been widely reported [86,87,88]. We used Targetscan miRNA as a prediction tool to assess which miRNAs may interact with their targets. A number of miRNAs are shown to putatively interact with Tucidinostat-induced DEGs. In addition, DL-sulforaphane treatment also altered the interaction between miRNAs and target mRNA. Subsequent experiments for functional validation of miRNA-target interactions in response to Tucidinostat and Dl-sulforaphane treatments are needed.

HDACs are divided into five groups based on sequence homology to the original yeast enzymes and domain organization. Among them, HDAC1, 2, 3, and 8 belong to Class I. Class IIa contains HDAC4, 5, 7, and 9. A previous study demonstrated that Tucidinostat targets Class I HDACs and sulforaphane attenuates class ILa HDACs and HDAC2 enzyme activities [89]. This study revealed several differences between Tucidinostat and DL-sulforaphane treatments in uLMS cells. (1) Tucidinostat showed a more potent inhibitory effect on cell proliferation than DL-sulforaphane. (2) Venn diagrams analysis demonstrated a distinct transcriptome pattern between Tucidinostat and DL-sulforaphane. (3) The expression levels of key genes such as p21 differed between Tucidinostat and DL-sulforaphane treatments. In addition, the enriched pathways and reprogramming differed between the two treatments. Tucidinostat showed similarity with other HDAC inhibitors in the transcriptome pattern, but DL-sulforaphane did not. From upset plot analysis, the groups of Tucidinostat and DL-sulforaphane contained a smaller number of common genes than either Tucidinostat/TP472 groups or DL-sulforaphane/TP472 groups, respectively. Since TP472 mainly targets BRD9, one may consider that Tucidinostat and DL-sulforaphane trigger the distinct expression pattern in uLMS cells markedly.

**Figure 17:**
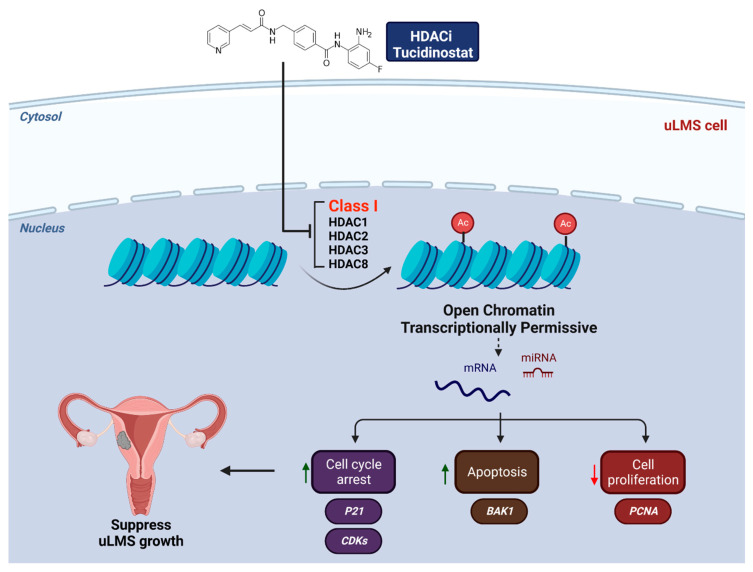
Experimental model. The model shows that Tucidinostat and DL-sulforaphane activate apoptosis, induce cell cycle arrest, induce miRNA-mediated gene regulation, and reprogram pro-oncogenic epigenome in uLMS cells. Note: Arabic numerals are the index for histone modifications.

Per our studies, we proposed a mechanism model for targeting HDACs in uLMS based on our novel findings that (1) Class I HDACs expression is dysregulated in uLMS tumors and cells, (2) targeting HDACs with Tucidinostat alters the uLMS phenotype with a decrease in cell proliferation and modulation of cell-cycle related genes, and increases the apoptosis process; (3) Tucidinostat and DL-sulforaphane reversed the phenotype of uLMS via different mechanism; (4) Class I HDACs constitutes a distinguished vulnerability in malignant uLMS, and HDAC inhibitors, such as Tucidinostat, alter key pathways and reprogram the oncogenic epigenome and miRNA network to suppress the uLMS phenotype (Figure 17).

The concept that preoperative therapy leads to the improvement of oncological results is widely approved for long-term survival in potentially curative cases and in those with metastatic diseases [90,91,92]. Unfortunately, no single preoperative test can reliably differentiate benign from malignant uterine disease [93]. Therefore, identifying biomarkers to differentiate malignant uLMS from benign UFs will help to initiate preoperative therapy to achieve a better outcome for patients with uLMS.

## 5. Conclusions

Our study demonstrated that uLMS tumors and cells exhibited an aberrant upregulation of class I HDAC proteins. Targeting HDACs in uLMS may impart beneficial effects in uLMS and provide a promising and novel strategy for treating patients with this aggressive uterine cancer. Furthermore, the present study provided a list of potential medications as inhibitors for EMT that can be used in combination with antineoplastic drugs to increase the sensitivity of tumor cells to improve responses to therapy in uLMS cells.

## Figures and Tables

**Table 1:** Antibodies used in this study.

Antibodies	Company	Catalog Number	Host	Application	Dilution	Size
HDAC1	Cell signaling	34589	Rabbit	WB	1:1000	62 kd
HDAC2	Cell signaling	57159	Rabbit	WB	1:1000	60 kd
HDAC3	Cell signaling	85057	Rabbit	WB	1:1000	49 kd
HDAC8	Abcam	ab187139	Rabbit	WB	1:1000	42 kd
PCNA	Abcam	Ab18197	Rabbit	WB	1:1000	29 kd
β-actin	Sigma	A5316	Mouse	WB	1:8000	42 kd
HDAC1	Abcam	Ab109411	Rabbit	IHC	1:50	
HDAC2	Abcam	Ab32117	Rabbit	IHC	1:500	
HDAC3	Abcam	Ab32369	Rabbit	IHC	1:4000	
Ki67	Abcam	ab15580	Rabbit	IHC	1: 3000	

## Data Availability

Raw FASTQ files were deposited in the NCBI Gene Expression Omnibus (GSE205777).

## Supplementary Materials

The following supporting information can be downloaded at: https://www.mdpi.com/article/10.3390/cells11233801/s1, Table S1: Tucidinostat drug similarity analysis; Table S2: DL-sulforaphane drug similarity analysis; Table S3: Candidate drugs that target the top 10 percent of EMT modules’ genes; Table S4: Drug candidates for EMT inhibition.
