# Supplementary material for: Targeting Class I Histone Deacetylases in Human Uterine Leiomyosarcoma
Source: Cells. 2022 Nov 27;11(23):3801. doi: 10.3390/cells11233801 (PMC9735512; doi:10.3390/cells11233801)
Supplement: Supplementary file 1 [file cells-11-03801-s001.zip › cells-2039253-supplementary.pdf]

Table S1. Tucidinostat drug similarity analysis

Rank	1-cos(alpha)	Perturbation	Cell.line	Dose	Time	Signature.URL
1	0.3386	BRD-K11663430	A375	10.0um	6.0h	https://maayanlab.cloud/L1000CDS2/meta?sig_id=CPC013_A375_6H:BRD-K11663430:10.0
2	0.3713	BRD-K60297835	HA1E	10.0um	6.0h	https://maayanlab.cloud/L1000CDS2/meta?sig_id=CPC019_HA1E_6H:BRD-K60297835:10.0
3	0.3733	PHA-665752	MCF7	10um	24h	https://maayanlab.cloud/L1000CDS2/meta?sig_id=LJP005_MCF7_24H:BRD-K95435023:10
4	0.3757	HDAC6 inhibitor ISOX	PL21	10.0um	6.0h	https://maayanlab.cloud/L1000CDS2/meta?sig_id=CPC006_PL21_6H:BRD-K69840642:10.0
5	0.3855	BRD-K97534490	MCF7	10.0um	6.0h	https://maayanlab.cloud/L1000CDS2/meta?sig_id=CPC009_MCF7_6H:BRD-K97534490:10.0
6	0.3953	BRD-K46373671	A375	10.0um	6.0h	https://maayanlab.cloud/L1000CDS2/meta?sig_id=CPC019_A375_6H:BRD-K46373671:10.0
7	0.3991	HDAC6 inhibitor ISOX	MCF7	10.0um	24.0h	https://maayanlab.cloud/L1000CDS2/meta?sig_id=CPC006_MCF7_24H:BRD-K69840642:10.0
8	0.4019	BRD-K87387614	MCF7	10.0um	24.0h	https://maayanlab.cloud/L1000CDS2/meta?sig_id=CPC019_MCF7_24H:BRD-K87387614:10.0
9	0.4083	QL-X-138	MCF10A	3.33um	24h	https://maayanlab.cloud/L1000CDS2/meta?sig_id=LJP006_MCF10A_24H:BRD-K72636697:3.33
10	0.409	mocetinostat	HA1E	1.11um	24h	https://maayanlab.cloud/L1000CDS2/meta?sig_id=LJP008_HA1E_24H:BRD-K16485616:1.11
11	0.4118	480743.cdx	A673	80.0um	6.0h	https://maayanlab.cloud/L1000CDS2/meta?sig_id=CPC006_A673_6H:BRD-K53903639:80.0
12	0.412	vorinostat	SNUC4	10.0um	6.0h	https://maayanlab.cloud/L1000CDS2/meta?sig_id=CPC006_SNUC4_6H:BRD-K81418486:10.0
13	0.4125	BRD-A25170757	MCF7	10.0um	24.0h	https://maayanlab.cloud/L1000CDS2/meta?sig_id=CPC010_MCF7_24H:BRD-A25170757:10.0
14	0.4146	WYE-125132	HME1	1.11um	24h	https://maayanlab.cloud/L1000CDS2/meta?sig_id=LJP006_HME1_24H:BRD-A45498368:1.11
15	0.4146	BRD-K52522949	HCC515	10.0um	6.0h	https://maayanlab.cloud/L1000CDS2/meta?sig_id=CPC020_HCC515_6H:BRD-K52522949:10.0
16	0.4158	entinostat	HA1E	0.37um	24h	https://maayanlab.cloud/L1000CDS2/meta?sig_id=LJP008_HA1E_24H:BRD-K77908580:0.37
17	0.4165	BRD-K55116708	A549	10.0um	24.0h	https://maayanlab.cloud/L1000CDS2/meta?sig_id=CPC019_A549_24H:BRD-K55116708:10.0
18	0.4192	wortmannin	HA1E	10.0um	6.0h	https://maayanlab.cloud/L1000CDS2/meta?sig_id=CPC013_HA1E_6H:BRD-A75409952:10.0
19	0.4236	GDC-0980	MCF10A	1.11um	24h	https://maayanlab.cloud/L1000CDS2/meta?sig_id=LJP005_MCF10A_24H:BRD-A18328003:1.11
20	0.4252	BI-2536	A375	0.37um	24h	https://maayanlab.cloud/L1000CDS2/meta?sig_id=LJP006_A375_24H:BRD-K64890080:0.37
21	0.4268	mocetinostat	HT29	0.37um	24h	https://maayanlab.cloud/L1000CDS2/meta?sig_id=LJP008_HT29_24H:BRD-K16485616:0.37
22	0.4277	rocilinostat	HT29	3.33um	24h	https://maayanlab.cloud/L1000CDS2/meta?sig_id=LJP008_HT29_24H:BRD-K82928847:3.33
23	0.4279	belinostat	MCF7	1.11um	24h	https://maayanlab.cloud/L1000CDS2/meta?sig_id=LJP008_MCF7_24H:BRD-K17743125:1.11
24	0.4292	BRD-K82580504	PC3	10.0um	24.0h	https://maayanlab.cloud/L1000CDS2/meta?sig_id=CPC009_PC3_24H:BRD-K82580504:10.0
25	0.4305	BRD-K77908580	SKB	10.0um	24.0h	https://maayanlab.cloud/L1000CDS2/meta?sig_id=CPC013_SKB_24H:BRD-K77908580:10.0
26	0.4306	vorinostat	LOVO	11.1um	6.0h	https://maayanlab.cloud/L1000CDS2/meta?sig_id=CPC006_LOVO_6H:BRD-K81418486:11.1
27	0.4344	NCGC00181736-02	HA1E	10.0um	6.0h	https://maayanlab.cloud/L1000CDS2/meta?sig_id=CPC007_HA1E_6H:BRD-K22210218:10.0
28	0.4364	GDC-0980	MCF10A	10um	24h	https://maayanlab.cloud/L1000CDS2/meta?sig_id=LJP005_MCF10A_24H:BRD-A18328003:10
29	0.4372	NU-7441	MCF7	10um	24h	https://maayanlab.cloud/L1000CDS2/meta?sig_id=LJP005_MCF7_24H:BRD-K00337317:10
30	0.4378	GDC-0980	MCF10A	3.33um	24h	https://maayanlab.cloud/L1000CDS2/meta?sig_id=LJP005_MCF10A_24H:BRD-A18328003:3.33
31	0.4385	WZ-3105	HT29	0.37um	24h	https://maayanlab.cloud/L1000CDS2/meta?sig_id=LJP005_HT29_24H:BRD-K92571446:0.37
32	0.4388	PI-103	HS578T	1.11um	24h	https://maayanlab.cloud/L1000CDS2/meta?sig_id=LJP006_HS578T_24H:BRD-K67868012:1.11
33	0.4391	480743.cdx	WSUDLCL2	80.0um	6.0h	https://maayanlab.cloud/L1000CDS2/meta?sig_id=CPC006_WSUDLCL2_6H:BRD-K53903639:80.
34	0.4411	AG 494	PC3	10.0um	24.0h	https://maayanlab.cloud/L1000CDS2/meta?sig_id=CPC001_PC3_24H:BRD-K81209512:10.0
35	0.4422	HY-10228	VCAP	10.0um	24.0h	https://maayanlab.cloud/L1000CDS2/meta?sig_id=CPC014_VCAP_24H:BRD-K99616396:10.0
36	0.4423	WYE-125132	MCF10A	3.33um	24h	https://maayanlab.cloud/L1000CDS2/meta?sig_id=LJP006_MCF10A_24H:BRD-A45498368:3.33
37	0.4432	HG-14-10-04	HT29	1.11um	24h	https://maayanlab.cloud/L1000CDS2/meta?sig_id=LJP007_HT29_24H:BRD-K20526256:1.11
38	0.4434	XMD16-144	MCF7	1.11um	24h	https://maayanlab.cloud/L1000CDS2/meta?sig_id=LJP005_MCF7_24H:BRD-U64521890:1.11
39	0.4437	Calcipotriol	PC3	10.0um	24.0h	https://maayanlab.cloud/L1000CDS2/meta?sig_id=CPC005_PC3_24H:BRD-K56429665:10.0
40	0.4444	H7270	PC3	10.0um	6.0h	https://maayanlab.cloud/L1000CDS2/meta?sig_id=CPC014_PC3_6H:BRD-A39646320:10.0
41	0.4452	mocetinostat	A375	0.37um	24h	https://maayanlab.cloud/L1000CDS2/meta?sig_id=LJP008_A375_24H:BRD-K16485616:0.37
42	0.4455	vorinostat	HA1E	10.0um	6.0h	https://maayanlab.cloud/L1000CDS2/meta?sig_id=CPC019_HA1E_6H:BRD-K81418486:10.0
43	0.4488	belinostat	HA1E	0.37um	24h	https://maayanlab.cloud/L1000CDS2/meta?sig_id=LJP008_HA1E_24H:BRD-K17743125:0.37
44	0.4503	AT-7519	HT29	1.11um	24h	https://maayanlab.cloud/L1000CDS2/meta?sig_id=LJP006_HT29_24H:BRD-K13390322:1.11
45	0.4505	linifanib	MCF7	10um	24h	https://maayanlab.cloud/L1000CDS2/meta?sig_id=LJP006_MCF7_24H:BRD-K99749624:10
46	0.4509	BRD-K06593056	MCF7	10.0um	6.0h	https://maayanlab.cloud/L1000CDS2/meta?sig_id=CPC015_MCF7_6H:BRD-K06593056:10.0
47	0.4515	BRD-K14200658	MCF7	10.0um	6.0h	https://maayanlab.cloud/L1000CDS2/meta?sig_id=CPC017_MCF7_6H:BRD-K14200658:10.0
48	0.4526	withaferin-a	MCF10A	1.11um	24h	https://maayanlab.cloud/L1000CDS2/meta?sig_id=LJP005_MCF10A_24H:BRD-K88378636:1.11
49	0.4528	BRD-K34092011	MCF7	10.0um	24.0h	https://maayanlab.cloud/L1000CDS2/meta?sig_id=CPC019_MCF7_24H:BRD-K34092011:10.0
50	0.4534	BI-2536	MCF10A	3.33um	24h	https://maayanlab.cloud/L1000CDS2/meta?sig_id=LJP006_MCF10A_24H:BRD-K64890080:3.33

Table S2. DL-sulforaphane drug similarity analysis

Rank	X1.cos.Î±.	Perturbation	Cell.line	Dose	Time	Signature.URL
1	0.2199	GSK-461364	HCC515	3.33um	24h	https://maayanlab.cloud/L1000CDS2/meta?sig_id=LJP008_HCC515_24H:BRD-K92428232:3.33
2	0.2547	AT-7519	MDAMB231	0.12um	24h	https://maayanlab.cloud/L1000CDS2/meta?sig_id=LJP006_MDAMB231_24H:BRD-K13390322:0.12
3	0.26	Ingenol 3, 20-dibenzoate	SNGM	10.0um	6.0h	https://maayanlab.cloud/L1000CDS2/meta?sig_id=CPC006_SNGM_6H:BRD-A52650764:10.0
4	0.2908	G3420	PHH	10.0um	24.0h	https://maayanlab.cloud/L1000CDS2/meta?sig_id=CPC014_PHH_24H:BRD-K28178212:10.0
5	0.2926	BRD-K09638361	HT29	10.0um	6.0h	https://maayanlab.cloud/L1000CDS2/meta?sig_id=CPC017_HT29_6H:BRD-K09638361:10.0
6	0.2969	diphenylcyclopropenone	PC3	10.0um	24.0h	https://maayanlab.cloud/L1000CDS2/meta?sig_id=CPC011_PC3_24H:BRD-K51730347:10.0
7	0.2992	BRD-K66792149	MDST8	11.1um	6.0h	https://maayanlab.cloud/L1000CDS2/meta?sig_id=CPC006_MDST8_6H:BRD-K66792149:11.1
8	0.3014	QL-XII-47	BT20	0.12um	24h	https://maayanlab.cloud/L1000CDS2/meta?sig_id=LJP006_BT20_24H:BRD-K99252563:0.12
9	0.3062	BRD-K96433222	VCAP	10.0um	6.0h	https://maayanlab.cloud/L1000CDS2/meta?sig_id=CPC013_VCAP_6H:BRD-K96433222:10.0
10	0.3092	withaferin-a	BT20	3.33um	24h	https://maayanlab.cloud/L1000CDS2/meta?sig_id=LJP005_BT20_24H:BRD-K88378636:3.33
11	0.3111	Rimcazole dihydrochloride	VCAP	10.0um	24.0h	https://maayanlab.cloud/L1000CDS2/meta?sig_id=CPC001_VCAP_24H:BRD-A63346720:10.0
12	0.3131	GW-843682X	SKBR3	3.33um	24h	https://maayanlab.cloud/L1000CDS2/meta?sig_id=LJP006_SKBR3_24H:BRD-K90382497:3.33
13	0.3146	QL-XII-47	HEPG2	1.11um	24h	https://maayanlab.cloud/L1000CDS2/meta?sig_id=LJP006_HEPG2_24H:BRD-K99252563:1.11
14	0.3179	BTB06091SC	PC3	10.0um	24.0h	https://maayanlab.cloud/L1000CDS2/meta?sig_id=CPC013_PC3_24H:BRD-K82685933:10.0
15	0.3238	PHA-793887	SKBR3	0.04um	24h	https://maayanlab.cloud/L1000CDS2/meta?sig_id=LJP006_SKBR3_24H:BRD-K64800655:0.04
16	0.3245	BRD-K72264770	MCF7	10.0um	6.0h	https://maayanlab.cloud/L1000CDS2/meta?sig_id=CPC012_MCF7_6H:BRD-K72264770:10.0
17	0.3259	BMS-345541	MDAMB231	3.33um	24h	https://maayanlab.cloud/L1000CDS2/meta?sig_id=LJP006_MDAMB231_24H:BRD-K13566078:3.33
18	0.3271	QL-XII-47	HEPG2	3.33um	24h	https://maayanlab.cloud/L1000CDS2/meta?sig_id=LJP006_HEPG2_24H:BRD-K99252563:3.33
19	0.3275	MLS-0390945.0001	VCAP	10.0um	6.0h	https://maayanlab.cloud/L1000CDS2/meta?sig_id=CPC013_VCAP_6H:BRD-K23027438:10.0
20	0.3301	HY-10161	VCAP	10.0um	24.0h	https://maayanlab.cloud/L1000CDS2/meta?sig_id=CPC013_VCAP_24H:BRD-K59369769:10.0
21	0.3304	ON-01910	A375	10um	24h	https://maayanlab.cloud/L1000CDS2/meta?sig_id=LJP009_A375_24H:BRD-K55187425:10
22	0.3307	QS 11	NOMO1	10.0um	6.0h	https://maayanlab.cloud/L1000CDS2/meta?sig_id=CPC006_NOMO1_6H:BRD-K02526760:10.0
23	0.3308	RACEPHEDRINE HYDROCHLORIDE	A549	10.0um	6.0h	https://maayanlab.cloud/L1000CDS2/meta?sig_id=CPC005_A549_6H:BRD-A54236247:10.0
24	0.3325	phorbol-12-myristate-13-acetate (PMA)	HEC108	10.0um	6.0h	https://maayanlab.cloud/L1000CDS2/meta?sig_id=CPC006_HEC108_6H:BRD-A15079084:10.0
25	0.3326	BRD-A10188456	HT29	10.0um	6.0h	https://maayanlab.cloud/L1000CDS2/meta?sig_id=CPC017_HT29_6H:BRD-A10188456:10.0
26	0.3333	phorbol-12-myristate-13-acetate (PMA)	SNGM	10.0um	6.0h	https://maayanlab.cloud/L1000CDS2/meta?sig_id=CPC006_SNGM_6H:BRD-A15079084:10.0
27	0.3344	BRD-K87426499	A549	10.0um	6.0h	https://maayanlab.cloud/L1000CDS2/meta?sig_id=CPC013_A549_6H:BRD-K87426499:10.0
28	0.3375	BRD-K26818574	MCF7	10.0um	24.0h	https://maayanlab.cloud/L1000CDS2/meta?sig_id=CPC012_MCF7_24H:BRD-K26818574:10.0
29	0.3379	BRD-K28916077	VCAP	10.0um	6.0h	https://maayanlab.cloud/L1000CDS2/meta?sig_id=CPC010_VCAP_6H:BRD-K28916077:10.0
30	0.3408	PNU 74654	H1299	80.0um	6.0h	https://maayanlab.cloud/L1000CDS2/meta?sig_id=CPC006_H1299_6H:BRD-K30707190:80.0
31	0.341	fluphenazine	VCAP	10.0um	6.0h	https://maayanlab.cloud/L1000CDS2/meta?sig_id=CPC005_VCAP_6H:BRD-K55127134:10.0
32	0.3421	BRD-K36354764	MCF7	10.0um	24.0h	https://maayanlab.cloud/L1000CDS2/meta?sig_id=CPC009_MCF7_24H:BRD-K36354764:10.0
33	0.3425	BRD-K90382497	MCF7	10.0um	24.0h	https://maayanlab.cloud/L1000CDS2/meta?sig_id=CPC006_MCF7_24H:BRD-K90382497:10.0
34	0.3431	BRD-K57309821	HT29	10.0um	6.0h	https://maayanlab.cloud/L1000CDS2/meta?sig_id=CPC019_HT29_6H:BRD-K57309821:10.0
35	0.3448	LASALOCID SODIUM	A375	10.0um	6.0h	https://maayanlab.cloud/L1000CDS2/meta?sig_id=CPC005_A375_6H:BRD-A72711497:10.0
36	0.345	2816	HA1E	10.0um	6.0h	https://maayanlab.cloud/L1000CDS2/meta?sig_id=CPC014_HA1E_6H:BRD-A52193669:10.0
37	0.3474	BRD-K24132293	PHH	10.0um	24.0h	https://maayanlab.cloud/L1000CDS2/meta?sig_id=CPC017_PHH_24H:BRD-K24132293:10.0
38	0.3485	BRD-K17140735	H1299	11.10000	6.0h	https://maayanlab.cloud/L1000CDS2/meta?sig_id=CPC006_H1299_6H:BRD-K17140735:11.1000003815
39	0.3496	NOCODAZOLE	MCF7	10.0um	6.0h	https://maayanlab.cloud/L1000CDS2/meta?sig_id=CPC004_MCF7_6H:BRD-K12539581:10.0
40	0.3503	BRD-K71935468	VCAP	10.0um	6.0h	https://maayanlab.cloud/L1000CDS2/meta?sig_id=CPC012_VCAP_6H:BRD-K71935468:10.0
41	0.3523	IMD 0354	MDST8	10.0um	6.0h	https://maayanlab.cloud/L1000CDS2/meta?sig_id=CPC006_MDST8_6H:BRD-K74305673:10.0
42	0.3562	Ingenol 3, 20-dibenzoate	MDST8	10.0um	6.0h	https://maayanlab.cloud/L1000CDS2/meta?sig_id=CPC006_MDST8_6H:BRD-A52650764:10.0
43	0.3566	BRD-K28916077	HA1E	10.0um	6.0h	https://maayanlab.cloud/L1000CDS2/meta?sig_id=CPC010_HA1E_6H:BRD-K28916077:10.0
44	0.357	BRD-K03816923	HEPG2	10.0um	6.0h	https://maayanlab.cloud/L1000CDS2/meta?sig_id=CPC018_HEPG2_6H:BRD-K03816923:10.0
45	0.3571	NP-009265	A549	10.0um	6.0h	https://maayanlab.cloud/L1000CDS2/meta?sig_id=CPC013_A549_6H:BRD-A57300602:10.0
46	0.3574	RHODOMYRTOXIN	PC3	10.0um	24.0h	https://maayanlab.cloud/L1000CDS2/meta?sig_id=CPC005_PC3_24H:BRD-K13725475:10.0
47	0.3605	masitinib	A375	10um	24h	https://maayanlab.cloud/L1000CDS2/meta?sig_id=LJP007_A375_24H:BRD-K71035033:10
48	0.3605	NCGC00182362-01	MCF7	10.0um	24.0h	https://maayanlab.cloud/L1000CDS2/meta?sig_id=CPC008_MCF7_24H:BRD-K07259155:10.0
49	0.3607	BRD-K46373671	PC3	10.0um	6.0h	https://maayanlab.cloud/L1000CDS2/meta?sig_id=CPC019_PC3_6H:BRD-K46373671:10.0
50	0.3607	Salermide	SNGM	120.0um	6.0h	https://maayanlab.cloud/L1000CDS2/meta?sig_id=CPC006_SNGM_6H:BRD-A67788537:120.0

Table S3. Candidate drugs that target the top 10 percent of EMT modules' genes

Gene	Drug	Interaction_types	Sources	PMIDS
IL6	SILTUXIMAB	antagonist|antibody|inhibitor	MyCancerGenome|ChemblInteractions|TTD	8823310
IL6	ETANERCEPT	NA	PharmGKB	24253594
IL6	IFOSFAMIDE	NA	NCI	9260581
IL6	RIBAVIRIN	NA	PharmGKB	NA
IL6	LEVOFLOXACIN	NA	NCI	12714806
IL6	GEMFIBROZIL	NA	NCI	8941582
IL6	NELFINAVIR	NA	NCI	15388451
IL6	SAQUINAVIR	NA	NCI	15388451
IL6	RITUXIMAB	NA	PharmGKB	26384320
IL6	INFLIXIMAB	NA	PharmGKB	24253594
IL6	CISPLATIN	NA	CIViC	21273582
IL6	FENOFIBRATE	NA	PharmGKB	16607077
IL6	FENTANYL	NA	NCI	9527747
IL6	ADALIMUMAB	NA	PharmGKB	24253594
IL6	INSULIN	NA	NCI	17392554
IL6	LINEZOLID	NA	NCI	14561977
IL6	METRONIDAZOLE	NA	NCI	12111578
KCNC1	DALFAMPRIDINE	blocker|antagonist	ChemblInteractions	16472864
KCNC1	GUANIDINE HYDROCHLORIDE	blocker	ChemblInteractions	NA
COL1A1	OCRIPLASMIN	NA	ChemblInteractions	NA
COL1A1	COLLAGENASE CLOSTRIDIUM HISTOLYTICUM	NA	ChemblInteractions|TEND	NA
PRKCG	MIDOSTAURIN	inhibitor	ChemblInteractions|TTD	NA
CACNA1G	TRIMETHADIONE	blocker|inhibitor	TdgClinicalTrial|ChemblInteractions|TEND|TTD	17291698|16171802|11752352|17139284|17016423
CACNA1G	ZONISAMIDE	inhibitor	TdgClinicalTrial|TEND	19557119|20001433|17762320|19948168|15511691|14965331|17139284|20025128|17016423|20502722|14704463|18433351
CACNA1G	GABAPENTIN	modulator	ChemblInteractions	NA
CACNA1G	METHSUXIMIDE	inhibitor|blocker	ChemblInteractions|TTD	2545161|11274992|19005061|11641441
CACNA1G	PARAMETHADIONE	blocker	ChemblInteractions|TTD	NA
CACNA1G	ETHOSUXIMIDE	blocker|inhibitor	TdgClinicalTrial|ChemblInteractions|TEND|TTD	11752352|11274992|19005061|11641441
CACNA1G	PREGABALIN	modulator	ChemblInteractions	NA
CACNA1G	GABAPENTIN ENACARBIL	modulator	ChemblInteractions	NA
CACNA1G	VERAPAMIL	inhibitor	TEND|TTD	16699084|21149638|19125880|11752352
CACNA1G	PHENSUXIMIDE	blocker	ChemblInteractions	NA
CACNA1G	BEPRIDIL HYDROCHLORIDE	blocker	ChemblInteractions	NA
CACNA1G	FLUNARIZINE	inhibitor	TdgClinicalTrial|TEND	19582593|11784784|17139284|17016423
IGF2	ETOPOSIDE	NA	NCI	15019164
IGF2	GEFITINIB	NA	NCI	16988945
IGF2	CABAZITAXEL	NA	CIViC	25670080
IGF2	GLUTAMINE	NA	NCI	14746834
IGF2	DOCETAXEL	NA	CIViC	25670080
IGF2	FLUOXETINE	NA	NCI	8265809
IGF2	GEMCITABINE	NA	CIViC	23741071

IGF2	ANASTROZOLE	NA	NCI	11983488
IGF2	BETAMETHASONE	NA	NCI	10495414
ATP1A3	DESLANOSIDE	inhibitor	ChemblInteractions	NA
ATP1A3	DIGITOXIN	inhibitor	ChemblInteractions	NA
ATP1A3	DIGOXIN	inhibitor	ChemblInteractions	NA
ATP1A3	ACETYLDIGITOXIN	inhibitor	ChemblInteractions	NA
SCN4B	ZONISAMIDE	inhibitor	TdgClinicalTrial	19557119|20001433|19948168|15511691|20025128|14704463|18433351
MMP2	CYCLOSPORINE	NA	NCI	12639820
MMP2	PRAVASTATIN	NA	NCI	15842807
MMP2	BEVACIZUMAB	NA	CIViC	26921265
MMP2	VINBLASTINE	NA	NCI	10590059
MMP2	FILGRASTIM	NA	NCI	11391619
MMP2	ZILEUTON	NA	DTC	24074025
MMP2	PACLITAXEL	NA	NCI	9174131
MMP2	SIMVASTATIN	NA	NCI	16436088
MMP2	LETROZOLE	NA	NCI	12569569
MMP2	STREPTOZOCIN	NA	NCI	9394952
MMP2	ACETAZOLAMIDE	NA	DTC	24074025
MMP2	DEFEROXAMINE	NA	DTC	24074025
MMP2	RAMIPRIL	NA	NCI	16166267
SLC6A1	COCAINE	NA	TEND	NA
SLC6A1	TIAGABINE	inhibitor	TdgClinicalTrial|TEND|TTD	16420077|16377242|20512624|17069932|11752352|18007567
SLC6A1	TIAGABINE HYDROCHLORIDE	inhibitor	ChemblInteractions	NA
CACNG 7	BEPRIDIL HYDROCHLORIDE	blocker	ChemblInteractions	NA
CACNG 7	PREGABALIN	modulator	ChemblInteractions	NA
CACNG 7	GABAPENTIN	modulator	ChemblInteractions	NA
CACNG 7	GABAPENTIN ENACARBIL	modulator	ChemblInteractions	NA
SCN4A	LEVOBUPIVACAINE HYDROCHLORIDE	blocker	ChemblInteractions	NA
SCN4A	RILUZOLE	blocker	ChemblInteractions	NA
SCN4A	PROPARACAINE HYDROCHLORIDE	blocker	ChemblInteractions	NA
SCN4A	PROCAINE HYDROCHLORIDE	blocker	ChemblInteractions	NA
SCN4A	CARBAMAZEPINE	blocker	ChemblInteractions	NA
SCN4A	LIDOCAINE HYDROCHLORIDE	blocker	ChemblInteractions	NA
SCN4A	BUPIVACAINE HYDROCHLORIDE	blocker	ChemblInteractions	NA
SCN4A	QUINIDINE SULFATE	blocker	ChemblInteractions	NA
SCN4A	DRONEDARONE HYDROCHLORIDE	blocker	ChemblInteractions	NA
SCN4A	ORPHENADRINE CITRATE	blocker	ChemblInteractions	NA
SCN4A	FOSPHENYTOIN SODIUM	blocker	ChemblInteractions	NA
SCN4A	PHENACEMIDE	blocker	ChemblInteractions	NA
SCN4A	RANOLAZINE	blocker	ChemblInteractions	NA
SCN4A	PROPOFOL	inhibitor	TdgClinicalTrial|TEND	18574460
SCN4A	ERLOSAMIDE	blocker	ChemblInteractions	NA
SCN4A	PROPOXYCAINE HYDROCHLORIDE	blocker	ChemblInteractions	NA

SCN4A	MEPHENYTOIN	blocker	ChemblInteractions	NA
SCN4A	PROCAINAMIDE HYDROCHLORIDE	blocker	ChemblInteractions	NA
SCN4A	ETIDOCAINE HYDROCHLORIDE	blocker	ChemblInteractions	NA
SCN4A	PHENYTOIN SODIUM	blocker	ChemblInteractions	NA
SCN4A	DYCLONINE HYDROCHLORIDE	blocker	ChemblInteractions	NA
SCN4A	ESLICARBAZEPINE ACETATE	blocker	ChemblInteractions	NA
SCN4A	TETRACAINE	blocker	ChemblInteractions	NA
SCN4A	INDECAINIDE HYDROCHLORIDE	blocker	ChemblInteractions	NA
SCN4A	OXCARBAZEPINE	blocker	ChemblInteractions	NA
SCN4A	PHENYTOIN	blocker	ChemblInteractions	NA
SCN4A	MEXILETINE HYDROCHLORIDE	blocker	ChemblInteractions	NA
SCN4A	BENOXINATE HYDROCHLORIDE	blocker	ChemblInteractions	NA
SCN4A	QUINIDINE POLYGALACTURONATE	blocker	ChemblInteractions	NA
SCN4A	BUPIVACAINE	blocker	ChemblInteractions	NA
SCN4A	PHENAZOPYRIDINE HYDROCHLORIDE	blocker	ChemblInteractions	NA
SCN4A	RUFINAMIDE	blocker	ChemblInteractions	NA
SCN4A	ESLICARBAZEPINE	blocker	ChemblInteractions	NA
SCN4A	PRIMIDONE	blocker	ChemblInteractions	NA
SCN4A	PRILOCAINE	blocker	ChemblInteractions	NA
SCN4A	QUINIDINE GLUCONATE	blocker	ChemblInteractions	NA
SCN4A	ETHOTOIN	blocker	ChemblInteractions	NA
SCN4A	SAFINAMIDE	NA	TdgClinicalTrial	NA
SCN4A	MEPIVACAINE HYDROCHLORIDE	blocker	ChemblInteractions	NA
SCN4A	ROPIVACAINE HYDROCHLORIDE	blocker	ChemblInteractions	NA
SCN4A	TOPIRAMATE	blocker	ChemblInteractions	NA
SCN4A	CHLOROPROCAINE HYDROCHLORIDE	blocker	ChemblInteractions	NA
SCN4A	ZONISAMIDE	inhibitor|blocker	TdgClinicalTrial|ChemblInteractions	19557119|20001433|19948168|15511691|20025128|14704463|18433351
SCN4A	DISOPYRAMIDE PHOSPHATE	blocker	ChemblInteractions	NA
SCN4A	PRILOCAINE HYDROCHLORIDE	blocker	ChemblInteractions	NA
SCN4A	ORPHENADRINE HYDROCHLORIDE	blocker	ChemblInteractions	NA
SCN4A	MORICIZINE HYDROCHLORIDE	blocker	ChemblInteractions	NA
SCN4A	LIDOCAINE	blocker	ChemblInteractions	14662728
SCN4A	HEXYLCAINE HYDROCHLORIDE	blocker	ChemblInteractions	NA
SCN4A	PROPAFENONE HYDROCHLORIDE	blocker	ChemblInteractions	NA
SCN4A	FOSPROPOFOL	NA	TEND	NA
SCN4A	LAMOTRIGINE	blocker	ChemblInteractions	NA
SCN4A	TOCAINIDE HYDROCHLORIDE	blocker	ChemblInteractions	NA
SCN4A	ARTICAINE HYDROCHLORIDE	blocker	ChemblInteractions	NA
SCN4A	MERETHOXYLLINE PROCAINE	blocker	ChemblInteractions	NA
SCN4A	TETRACAINE HYDROCHLORIDE	blocker	ChemblInteractions	NA
CXCL8	VERAPAMIL	NA	NCI	2686646
CXCL8	TRETINOIN	NA	NCI	8900181
CXCL8	ASPIRIN	NA	NCI	12576442

CXCL8	ACETAMINOPHEN	NA	NCI	15878691
CXCL8	CLARITHROMYCIN	NA	NCI	12003967
CXCL8	PYROGALLOL	NA	DTC	NA
CXCL8	TALC	NA	NCI	17000556
CXCL8	RETINOL	NA	DTC	NA
CXCL8	HYDROQUINONE	NA	DTC|NCI	17118622
CXCL8	METHIMAZOLE	NA	NCI	11453524
CXCL8	CEFTRIAXONE	NA	NCI	8011012
CXCL8	DANAZOL	NA	NCI	16161451
CXCL8	DACARBAZINE	NA	DTC	NA
CXCL8	RIBAVIRIN	NA	DTC	NA
CXCL8	ALPRAZOLAM	NA	NCI	12218154
CXCL8	CIDOFOVIR	NA	NCI	10630964
CXCL8	IBUPROFEN	NA	TTD	NA
CXCL8	PENTOXIFYLLINE	NA	NCI	12576442
CXCL8	BEVACIZUMAB	NA	PharmGKB	23584701
CXCL8	DIPYRIDAMOLE	NA	NCI	10660968
CXCL8	SUNITINIB	NA	PharmGKB	26387812
CXCL8	CETUXIMAB	NA	NCI	10614716|15908664|10037173
CXCL8	LEFLUNOMIDE	NA	NCI	10902750
CXCL8	OMEPRAZOLE	NA	NCI	17122965
CXCL8	PAMIDRONIC ACID	NA	NCI	12006522
CXCL8	NAPROXEN	NA	NCI	11852880
CXCL8	FENTANYL	NA	NCI	9527747
CXCL8	FOSCARNET	NA	NCI	10630964
CXCL8	LANSOPRAZOLE	NA	NCI	17122965
CXCL8	COLCHICINE	NA	DTC	NA
CXCL8	PACLITAXEL	NA	NCI	9271387
CXCL8	MIDAZOLAM	NA	NCI	9620522
COL5A1	OCRIPLASMIN	NA	ChemblInteractions	NA
COL5A1	COLLAGENASE CLOSTRIDIUM HISTOLYTICUM	NA	ChemblInteractions	NA
KCNB1	GUANIDINE HYDROCHLORIDE	blocker	ChemblInteractions	NA
KCNB1	DALFAMPRIDINE	antagonist|blocker	ChemblInteractions	16472864
KCNJ2	DRONEDARONE HYDROCHLORIDE	blocker	ChemblInteractions	NA
ERBB3	OSIMERTINIB MESYLATE	inhibitor	ChemblInteractions	NA
ERBB3	CARBOPLATIN	NA	PharmGKB	30071039
ERBB3	TRASTUZUMAB	NA	DoCM|CIViC|PharmGKB	23680147|25953157|30071039
ERBB3	ERLOTINIB	NA	PharmGKB	NA
ERBB3	DACOMITINIB	inhibitor	ChemblInteractions|MyCancerGenomeClinicalTrial	NA
ERBB3	PERTUZUMAB	NA	DoCM|CIViC	23680147|26206558|25216528
ERBB3	DOCETAXEL	NA	PharmGKB	30071039
ERBB3	VANDETANIB	inhibitor	ChemblInteractions	NA
ERBB3	CETUXIMAB	NA	CIViC	25520391

ERBB3	AFATINIB	NA	CIViC	27044931|24685132
ERBB3	GEFITINIB	inhibitor	CIViC|MyCancerGenomeClinicalTrial|PharmGKB	24685132
ERBB3	LAPATINIB	NA	DoCM|CIViC|PharmGKB	23680147|25953157|25398453
ERBB3	TRAMETINIB	NA	CIViC	25952648
MMP1	RIBAVIRIN	NA	NCI	16699498
MMP1	COLLAGENASE CLOSTRIDIUM HISTOLYTICUM	NA	TdgClinicalTrial	NA
MMP1	MEDROXYPROGESTERONE ACETATE	NA	NCI	9436888
MMP1	LAMIVUDINE	NA	NCI	15309715
MMP1	PENTOSAN POLYSULFATE SODIUM	NA	NCI	1384503
MMP1	LEFLUNOMIDE	NA	NCI	16762150
MMP1	TRIAMCINOLONE	NA	NCI	12123742
MMP1	DOXYCYCLINE CALCIUM	inhibitor	ChemblInteractions	NA
MMP1	DOXYCYCLINE	inhibitor	ChemblInteractions	NA
MMP1	SIROLIMUS	NA	NCI	16914544
MMP1	LEUPROLIDE ACETATE	NA	NCI	9433928
MMP1	HYDROCORTISONE	NA	NCI	7592884

Table S4. Drug candidates for EMT inhibition

Rank	1-cos(Alpha)	Perturbation	Perturbation.LIFE.URL	Perturbation.PubChem.URL	Perturbation.DrugBank.URL	Cell.line	Dose	Time
1	1.5159	CX-5461	http://life.ccs.miami.edu/life/summary?mode=SmallMolecule&source=BROAD&input=BRD-K12787259	None	None	PC3	0.37um	24h
2	1.4859	BRD-K96799727	http://life.ccs.miami.edu/life/summary?mode=SmallMolecule&source=BROAD&input=BRD-K96799727	http://pubchem.ncbi.nlm.nih.gov/summary/summary.cgi?cid=327653	None	HEC108	10.0um	6.0h
3	1.4721	PD-0325901	http://life.ccs.miami.edu/life/summary?mode=SmallMolecule&source=BROAD&input=BRD-K49865102	http://pubchem.ncbi.nlm.nih.gov/summary/summary.cgi?cid=9826528	None	MCF7	1.11um	3h
4	1.4486	VEGF Receptor 2 Kinase Inhibitor IV	http://life.ccs.miami.edu/life/summary?mode=SmallMolecule&source=BROAD&input=BRD-K61737877	http://pubchem.ncbi.nlm.nih.gov/summary/summary.cgi?cid=5329468	None	VCAP	10.0um	24.0h
5	1.4398	motesanib	http://life.ccs.miami.edu/life/summary?mode=SmallMolecule&source=BROAD&input=BRD-K99616396	http://pubchem.ncbi.nlm.nih.gov/summary/summary.cgi?cid=11667893	None	MCF7	10um	24h
6	1.4396	BRD-K08448573	http://life.ccs.miami.edu/life/summary?mode=SmallMolecule&source=BROAD&input=BRD-K08448573	http://pubchem.ncbi.nlm.nih.gov/summary/summary.cgi?cid=44511133	None	HEPG2	10.0um	6.0h
7	1.4285	BW-B 70C	http://life.ccs.miami.edu/life/summary?mode=SmallMolecule&source=BROAD&input=BRD-A68891053	http://pubchem.ncbi.nlm.nih.gov/summary/summary.cgi?cid=5353454	None	VCAP	10.0um	24.0h
8	1.4235	Ch 55	http://life.ccs.miami.edu/life/summary?mode=SmallMolecule&source=BROAD&input=BRD-K51290057	http://pubchem.ncbi.nlm.nih.gov/summary/summary.cgi?cid=6184667	None	HT29	10.0um	6.0h
9	1.4233	NOCODAZOLE	http://life.ccs.miami.edu/life/summary?mode=SmallMolecule&source=BROAD&input=BRD-K12539581	http://pubchem.ncbi.nlm.nih.gov/summary/summary.cgi?cid=4122	None	HT29	10.0um	6.0h
10	1.4209	SB 225002	http://life.ccs.miami.edu/life/summary?mode=SmallMolecule&source=BROAD&input=BRD-K61323504	http://pubchem.ncbi.nlm.nih.gov/summary/summary.cgi?cid=3854666	None	VCAP	10.0um	24.0h
11	1.4203	BRD-K67860401	http://life.ccs.miami.edu/life/summary?mode=SmallMolecule&source=BROAD&input=BRD-K67860401	http://pubchem.ncbi.nlm.nih.gov/summary/summary.cgi?cid=448014	None	A549	10.0um	24.0h
12	1.4178	Phensuximide	http://life.ccs.miami.edu/life/summary?mode=SmallMolecule&source=BROAD&input=BRD-A18043272	http://pubchem.ncbi.nlm.nih.gov/summary/summary.cgi?cid=6839	http://www.drugbank.ca/drugs/DB00832	MCF7	10.0um	6.0h
13	1.41	MLN4924	http://life.ccs.miami.edu/life/summary?mode=SmallMolecule&source=BROAD&input=BRD-K67844266	http://pubchem.ncbi.nlm.nih.gov/summary/summary.cgi?cid=16720766	None	HEPG2	11.1um	6.0h

14	1.4067	MLS-0106435.0004	http://life.ccs.miami.edu/life/summary?mode=SmallMolecule&source=BROAD&input=BRD-K67118123	http://pubchem.ncbi.nlm.nih.gov/summary/summary.cgi?cid=25199560	None	VCAP	10.0um	24.0h
15	1.4063	Azlocillin sodium salt	http://life.ccs.miami.edu/life/summary?mode=SmallMolecule&source=BROAD&input=BRD-K73437736	http://pubchem.ncbi.nlm.nih.gov/summary/summary.cgi?cid=6560164	None	PC3	10.0um	6.0h
16	1.4051	BRD-K14711204	http://life.ccs.miami.edu/life/summary?mode=SmallMolecule&source=BROAD&input=BRD-K14711204	http://pubchem.ncbi.nlm.nih.gov/summary/summary.cgi?cid=50904528	None	HT29	10.0um	6.0h
17	1.4039	BRD-K18163752	http://life.ccs.miami.edu/life/summary?mode=SmallMolecule&source=BROAD&input=BRD-K18163752	http://pubchem.ncbi.nlm.nih.gov/summary/summary.cgi?cid=24030598	None	HEPG2	10.0um	6.0h
18	1.4012	Emetine Dihydrochloride Hydrate (74)	http://life.ccs.miami.edu/life/summary?mode=SmallMolecule&source=BROAD&input=BRD-K01976263	http://pubchem.ncbi.nlm.nih.gov/summary/summary.cgi?cid=11957493	None	A375	0.63um	6.0h
19	1.4001	ODQ	http://life.ccs.miami.edu/life/summary?mode=SmallMolecule&source=BROAD&input=BRD-K26015241	http://pubchem.ncbi.nlm.nih.gov/summary/summary.cgi?cid=1456	None	VCAP	10.0um	24.0h
20	1.3975	Xaliproden hydrochloride	http://life.ccs.miami.edu/life/summary?mode=SmallMolecule&source=BROAD&input=BRD-K88358234	http://pubchem.ncbi.nlm.nih.gov/summary/summary.cgi?cid=128919	None	VCAP	10.0um	6.0h
21	1.3973	ST4049616	http://life.ccs.miami.edu/life/summary?mode=SmallMolecule&source=BROAD&input=BRD-K18587499	http://pubchem.ncbi.nlm.nih.gov/summary/summary.cgi?cid=6602526	None	A549	10.0um	6.0h
22	1.3971	BRD-K23657553	http://life.ccs.miami.edu/life/summary?mode=SmallMolecule&source=BROAD&input=BRD-K23657553	http://pubchem.ncbi.nlm.nih.gov/summary/summary.cgi?cid=44500949	None	A549	10.0um	6.0h
23	1.3948	CYCLOHEXIMIDE	http://life.ccs.miami.edu/life/summary?mode=SmallMolecule&source=BROAD&input=BRD-K36055864	http://pubchem.ncbi.nlm.nih.gov/summary/summary.cgi?cid=6197	None	HT29	10.0um	6.0h
24	1.3944	VU0365114-2	http://life.ccs.miami.edu/life/summary?mode=SmallMolecule&source=BROAD&input=BRD-K37456065	http://pubchem.ncbi.nlm.nih.gov/summary/summary.cgi?cid=45281794	None	HT29	10.0um	6.0h
25	1.394	NCGC00180995-01	http://life.ccs.miami.edu/life/summary?mode=SmallMolecule&source=BROAD&input=BRD-K89563433	http://pubchem.ncbi.nlm.nih.gov/summary/summary.cgi?cid=44142113	None	VCAP	10.0um	24.0h
26	1.3919	BRD-K72029282	http://life.ccs.miami.edu/life/summary?mode=SmallMolecule&source=BROAD&input=BRD-K72029282	http://pubchem.ncbi.nlm.nih.gov/summary/summary.cgi?cid=4912	None	NPC	10.0um	24.0h
27	1.3917	CHIR-99021	http://life.ccs.miami.edu/life/summary?mode=SmallMolecule&source=BROAD&input=BRD-K16189898	http://pubchem.ncbi.nlm.nih.gov/summary/summary.cgi?cid=9956119	None	MDAMB231	3.33um	24h
28	1.3913	NCGC00238427-01	http://life.ccs.miami.edu/life/summary?mode=SmallMolecule&source=BROAD&input=BRD-A38793261	http://pubchem.ncbi.nlm.nih.gov/summary/summary.cgi?cid=46943339	None	HT29	10.0um	6.0h
29	1.3892	SARMENTOGENIN	http://life.ccs.miami.edu/life/summary?mode=SmallMolecule&source=BROAD&input=BRD-A89434049	http://pubchem.ncbi.nlm.nih.gov/summary/summary.cgi?cid=6708600	None	VCAP	10.0um	6.0h
30	1.3887	S1527	http://life.ccs.miami.edu/life/summary?mode=SmallMolecule&source=BROAD&input=BRD-K10916986	http://pubchem.ncbi.nlm.nih.gov/summary/summary.cgi?cid=73707409	None	PHH	10.0um	24.0h
31	1.3882	BRD-K68143200	http://life.ccs.miami.edu/life/summary?mode=SmallMolecule&source=BROAD&input=BRD-K68143200	http://pubchem.ncbi.nlm.nih.gov/summary/summary.cgi?cid=262093	None	RMUGS	10.0um	6.0h
32	1.3855	BRD-A93393712	http://life.ccs.miami.edu/life/summary?mode=SmallMolecule&source=BROAD&input=BRD-A93393712	http://pubchem.ncbi.nlm.nih.gov/summary/summary.cgi?cid=51003685	None	HT29	10.0um	6.0h
33	1.3835	PKCbeta inhibitor	http://life.ccs.miami.edu/life/summary?mode=SmallMolecule&source=BROAD&input=BRD-K89687904	http://pubchem.ncbi.nlm.nih.gov/summary/summary.cgi?cid=6419755	None	VCAP	10.0um	24.0h
34	1.3822	BRD-K75430629	http://life.ccs.miami.edu/life/summary?mode=SmallMolecule&source=BROAD&input=BRD-K75430629	http://pubchem.ncbi.nlm.nih.gov/summary/summary.cgi?cid=9908783	None	HT29	10.0um	6.0h
35	1.3812	BRD-K94991378	http://life.ccs.miami.edu/life/summary?mode=SmallMolecule&source=BROAD&input=BRD-K94991378	http://pubchem.ncbi.nlm.nih.gov/summary/summary.cgi?cid=6731789	None	MCF7	10.0um	6.0h
36	1.3807	BRD-K94991378	http://life.ccs.miami.edu/life/summary?mode=SmallMolecule&source=BROAD&input=BRD-K94991378	http://pubchem.ncbi.nlm.nih.gov/summary/summary.cgi?cid=6731789	None	HA1E	10.0um	6.0h
37	1.3803	BRD-K26818574	http://life.ccs.miami.edu/life/summary?mode=SmallMolecule&source=BROAD&input=BRD-K26818574	http://pubchem.ncbi.nlm.nih.gov/summary/summary.cgi?cid=25150857	None	HT29	10.0um	6.0h
38	1.3795	BRD-K53932786	http://life.ccs.miami.edu/life/summary?mode=SmallMolecule&source=BROAD&input=BRD-K53932786	http://pubchem.ncbi.nlm.nih.gov/summary/summary.cgi?cid=781660	None	MCF7	10.0um	6.0h
39	1.378	F3103-0039	http://life.ccs.miami.edu/life/summary?mode=SmallMolecule&source=BROAD&input=BRD-A93893742	http://pubchem.ncbi.nlm.nih.gov/summary/summary.cgi?cid=2203170	None	HEPG2	10.0um	6.0h
40	1.3776	BRD-K51318897	http://life.ccs.miami.edu/life/summary?mode=SmallMolecule&source=BROAD&input=BRD-K51318897	http://pubchem.ncbi.nlm.nih.gov/summary/summary.cgi?cid=3334	None	HT29	10.0um	6.0h
41	1.3769	CT-INCB	http://life.ccs.miami.edu/life/summary?mode=SmallMolecule&source=BROAD&input=BRD-K53972329	http://pubchem.ncbi.nlm.nih.gov/summary/summary.cgi?cid=25126798	None	HEPG2	10.0um	6.0h

42	1.3745	H-5832	http://life.ccs.miami.edu/life/summary?mode=SmallMolecule&source=BROAD&input=BRD-A32161980	http://pubchem.ncbi.nlm.nih.gov/summary/summary.cgi?cid=73707428	None	VCAP	10.0um	24.0h
43	1.3742	CP 94253 hydrochloride	http://life.ccs.miami.edu/life/summary?mode=SmallMolecule&source=BROAD&input=BRD-K33860217	http://pubchem.ncbi.nlm.nih.gov/summary/summary.cgi?cid=4029677	None	HCC515	10.0um	24.0h
44	1.3738	Pravastatin sodium salt	http://life.ccs.miami.edu/life/summary?mode=SmallMolecule&source=BROAD&input=BRD-K60511616	http://pubchem.ncbi.nlm.nih.gov/summary/summary.cgi?cid=54687	http://www.drugbank.ca/drugs/DB00175	VCAP	10.0um	6.0h
45	1.3738	BW-B 70C	http://life.ccs.miami.edu/life/summary?mode=SmallMolecule&source=BROAD&input=BRD-A68891053	http://pubchem.ncbi.nlm.nih.gov/summary/summary.cgi?cid=5353454	None	VCAP	10.0um	6.0h
46	1.3722	BRD-A25775766	http://life.ccs.miami.edu/life/summary?mode=SmallMolecule&source=BROAD&input=BRD-A25775766	http://pubchem.ncbi.nlm.nih.gov/summary/summary.cgi?cid=6338099	None	HT29	10.0um	6.0h
47	1.372	PX12	http://life.ccs.miami.edu/life/summary?mode=SmallMolecule&source=BROAD&input=BRD-A56592690	http://pubchem.ncbi.nlm.nih.gov/summary/summary.cgi?cid=219104	None	RMGI	30.0um	6.0h
48	1.3715	NCGC00188721-01	http://life.ccs.miami.edu/life/summary?mode=SmallMolecule&source=BROAD&input=BRD-K02381115	http://pubchem.ncbi.nlm.nih.gov/summary/summary.cgi?cid=50897779	None	HT29	10.0um	6.0h
49	1.3713	BRD-K98173387	http://life.ccs.miami.edu/life/summary?mode=SmallMolecule&source=BROAD&input=BRD-K98173387	http://pubchem.ncbi.nlm.nih.gov/summary/summary.cgi?cid=44807133	None	VCAP	10.0um	24.0h
50	1.3712	BRD-K77987382	http://life.ccs.miami.edu/life/summary?mode=SmallMolecule&source=BROAD&input=BRD-K77987382	http://pubchem.ncbi.nlm.nih.gov/summary/summary.cgi?cid=4030	http://www.drugbank.ca/drugs/DB00643	HEPG2	10.0um	6.0h
